# RNA Mimicry by the Fap7 Adenylate Kinase in Ribosome Biogenesis

**DOI:** 10.1371/journal.pbio.1001860

**Published:** 2014-05-13

**Authors:** Jérôme Loc'h, Magali Blaud, Stéphane Réty, Simon Lebaron, Patrick Deschamps, Joseph Bareille, Julie Jombart, Julien Robert-Paganin, Lila Delbos, Florian Chardon, Elodie Zhang, Clément Charenton, David Tollervey, Nicolas Leulliot

**Affiliations:** 1 Laboratoire de Cristallographie et RMN Biologiques, UMR CNRS 8015, Université Paris Descartes, Faculté de Pharmacie, Sorbonne Paris Cité, Paris, France; 2 Wellcome Trust Centre for Cell Biology, University of Edinburgh, Edinburgh, Scotland; Brandeis University, United States of America

## Abstract

During biogenesis of the 40S and 60S ribosomal subunits, the pre-40S particles are exported to the cytoplasm prior to final cleavage of the 20S pre-rRNA to mature 18S rRNA. Amongst the factors involved in this maturation step, Fap7 is unusual, as it both interacts with ribosomal protein Rps14 and harbors adenylate kinase activity, a function not usually associated with ribonucleoprotein assembly. Human hFap7 also regulates Cajal body assembly and cell cycle progression via the p53–MDM2 pathway. This work presents the functional and structural characterization of the Fap7–Rps14 complex. We report that Fap7 association blocks the RNA binding surface of Rps14 and, conversely, Rps14 binding inhibits adenylate kinase activity of Fap7. In addition, the affinity of Fap7 for Rps14 is higher with bound ADP, whereas ATP hydrolysis dissociates the complex. These results suggest that Fap7 chaperones Rps14 assembly into pre-40S particles via RNA mimicry in an ATP-dependent manner. Incorporation of Rps14 by Fap7 leads to a structural rearrangement of the platform domain necessary for the pre-rRNA to acquire a cleavage competent conformation.

## Abstract 2

The structure of a ribosome assembly factor in complex bound to a ribosomal protein uncovers a chaperoning function that uses RNA mimicry and is regulated by ATP hydrolysis.

## Author Summary

Ribosomes are the cellular machines responsible for all protein synthesis. In eukaryotes, the assembly of ribosomes from their protein and RNA components is extremely complicated and involves more than 200 nonribosomal factors—three times the number of proteins in the mature complex. Among these factors, the Fap7 protein is particularly intriguing because it interacts with the small subunit ribosomal protein Rps14 and it exhibits adenylate kinase activity—a molecular function more commonly associated with regulating ATP/ADP levels than assembling protein–RNA complexes. Combining structural and biochemical analysis of the Rps14–Fap7 complex, we show that Fap7 uses protein side chains to mimic RNA contacts, rendering the interaction of Rps14 with ribosomal RNA or with Fap7 competitive and mutually exclusive. Once bound, Rps14 blocks the substrate-binding cavity of Fap7, and ATP hydrolysis will then break the Fap7–Rps14 complex apart. At the same time, the ribosome structure at the location where Rps14 binds is disrupted when the Fap7/Rps14 complex is formed, and this process is regulated by ATP binding and hydrolysis. Our model is thus that Fap7 temporarily removes Rps14 from the ribosome to enable a conformational change of the ribosomal RNA that is needed for the final maturation step of the small ribosomal subunit.

## Introduction

Over 200 preribosomal factors are involved in the maturation of ribosomes. Most of these factors are essential for cell survival, but their precise molecular functions remain elusive (for reviews, see [1]–[3]). One of the last steps of maturation of the small subunit of the ribosome is the cytoplasmic cleavage of the 20S pre-rRNA at site D to generate 18S rRNA. This cleavage is carried out by the endonuclease Nob1 in 80S-like complexes composed of pre-40S particles and mature 60S [4],[5]. Cleavage also requires multiple pre-40S factors including the Nob1 binding protein Pno1/Dim2, the methyltransferase Dim1, the export factors Enp1 and Ltv1, and several NTPases including the Rio1 and Rio2 protein kinases, the Prp43 helicase and its cofactor Pfa1, the GTPase-related factor Tsr1, and the Fap7 NTPase.

The locations of maturation factors in the late pre-40S particles is emerging from *in vivo* RNA binding (CRAC), cryo-EM, and crystallographic studies on preribosomal particles [6]–[10], but detailed understanding of their functions remains limited. Amongst late pre-40S factors, the function of Fap7 is especially intriguing. Human hFap7 (also called hCINAP or AK6) bears structural homology to adenylate kinases (AKs) and harbors a broad AK activity [11],[12]. AKs catalyze the reversible transfer of the γ phosphate of adenosine nucleotide triphosphate (ATP) to an adenosine mono-phosphate (AMP), forming two molecules of adenosine di-phosphate (ADP). AKs play important roles in nucleotide metabolism [13], but the link between this enzymatic activity and ribonucleoprotein (RNP) assembly is enigmatic.

In yeast, yFap7 is necessary for the late cytoplasmic maturation steps of the 40S particle, and strictly required for the cleavage at site D [5],[14]. However, the association of yFap7 with the pre-40S particles is either weak or very transient [5],[14]. In the absence of yFap7, pre-40S subunits accumulate in 80S-like particles, containing 20S pre-rRNA. An active site mutant in yFap7 has the same phenotype, demonstrating a requirement for AK activity [5],[14]. The catalytic activity of hFap7 is also important in the assembly and/or stability of Cajal bodies [12],[15],[16], nuclear domains involved in the maturation of small nuclear RNP (snRNP) particles [17].

Fap7 forms a complex with Rps14 that is conserved between humans, yeast, and archea [14],[18],[19]. Rps14 is a component of the platform domain of the small subunit, and its C terminus forms an extended structure rich in basic residues, which binds the rRNA close to Helix 45 and site D. This C-terminal extension is essential for D site cleavage, and point mutations in yRps14 show effects on ribosome biogenesis similar to yFap7 depletion [14],[20]. Human hRps14 has at least two links to ribosomopathies and to cancer. Haploinsufficiency of hRps14 is a causal factor in myelodysplastic syndrome (MDS) and 5q syndrome, a genetic disorder related to Diamond Blackfan anemia that leads to severe anaemia, macrocytosis, and an increased risk of leukaemia [21],[22]. Additionally, hRps14 regulates the MDM2–p53 pathway by directly binding the acidic domain of MDM2 [23]. This interaction prevents MDM2 from targeting p53 for degradation, resulting in p53 activation and cell cycle arrest. This activity links deregulation of ribosome biogenesis in response to nucleolar stress to the inhibition of cell cycle progression. hFap7 is also an essential regulator of the hRps14–HDM2–p53 pathway by affecting the interaction between hRps14 and HDM2 [18].

To decipher the function of Fap7 in ribosome biogenesis, we functionally and structurally characterized the Fap7 protein alone and in complex with Rps14 and nucleotides. The combination of structural studies, enzymatic assays, RNA binding studies, and *in vitro* D site cleavage assays on purified preribosomes uncovered the function of Fap7 within pre-40S ribosomes.

## Results

### Overall Structure of the Fap7–Rps14 Complex

**Figure 1:**
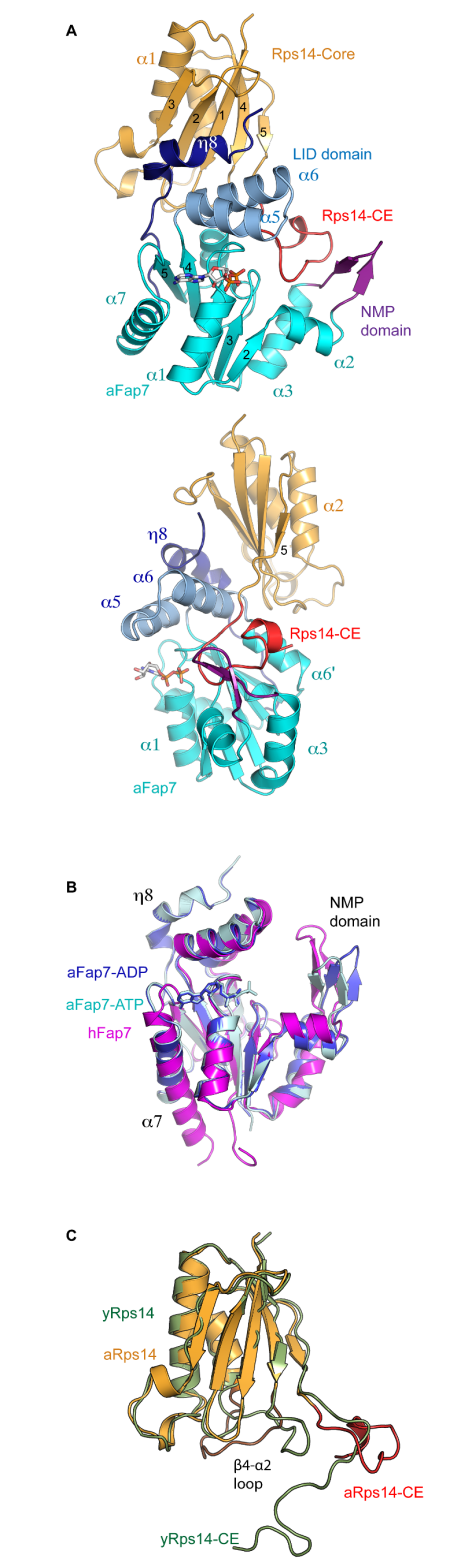
Structure of the aFap7–aRps14 complex. (A) Cartoon representation of the aFap7–aRps14 complex in complex with ADP. aFap7 is represented in blue/purple shades and Rps14 in orange for the CORE domain and red for the C-terminal extension. (B) Superposition of the ADP (light blue) and ATP (dark blue) aFap7 structure with the hFap7 in complex with ADP-Pi (magenta, PDB 3iil). (C) Superposition of aRps14 in complex with aFap7 (orange of the core domain, red for the Rps14-CE, and brown for the β4–α2 loop), with yRps14 in complex with the ribosome (green)

When expressed alone, yRps14 showed poor solubility. We therefore designed polycistronic vectors to co-express Fap7 and Rps14 from the yeast *Saccharomyces cerevisiae* (yFap7/yRps14) and the archaeon *Pyrococcus abyssi* (aFap7/aRps14). A His6 affinity tag was fused to the N terminus of Fap7 for a first purification step by immobilized Nickel metal ion affinity chromatography (Ni-IMAC). Excess-free Fap7 was separated from the Fap7–Rps14 complex by gel filtration. Two crystal forms were obtained for the archaeal aFap7–aRps14 complex bound to ADP/Mg^2+^ and ATP/Mg^2+^, which diffracted to 2.1 Å and 2.4 Å, respectively. Phasing was performed by single anomalous diffraction on the ADP/Mg^2+^ crystal form with platinum-derived crystals diffracting to 4.0 Å (see phasing and refinement statistics in Table 1). Four copies of the Fap7–Rps14 complex are present in the asymmetric unit in both space groups, revealing a 1∶1 complex of aFap7 with aRps14 (Figure 1A).

**Table 1:** Data collection and refinement statistics.

Ligands	ADP	ATP
Wavelength (Å)	0.98	0.97
Resolution (Å)	2.12 (2.25–2.12)	2.45 (2.60–2.45)
Space group	P21	P21212
Cell dimensions (Å)	67.0, 139.7, 83.1	125.2, 142.9, 84.0
α, β, γ (°)	90, 105.46, 90	90, 90, 90
No. of observed reflections	312,402 (48,359)	395,688 (59,519)
No. of unique reflections	82,662 (13,401)	55,229 (8,413)
Mosaicity (°)	0.162	0.146
Multiplicity	3.8	7.2
I/σ(I)	13.90 (2.36)	12.82 (2.63)
Completeness (%)	99.4 (97.3)	99.1 (94.5)
Rmerge (%)	6.8 (66.3)	12.6 (87.5)
Matthews coefficient VM (Å^3^ Da^−1^)	2.57	2.57
Solvent content (%)	52.21	52.24
Molecules per ASU	8	8
Refinement		
Rwork (Rfree)	0.17 (0.21)	0.19 (0.23)
RMS deviation in bond lengths (Å)	0.008	0.008
RMS deviation in bond angles (°)	1.238	1.317
Ramachandran (%)		
Favored	98.8	98.5
Allowed	1.2	1.5

The structure of bound aFap7 is similar to the previously reported human homologue hFap7, with 2.1 Å r.m.s.d. for 30% identity (Figure 1B, alignment in Figure S1A). All structural elements of the CORE domain (cyan in Figure 1A) adopt the canonical fold common to AKs [24]. aFap7 also contains the specific structures of the Fap7 AK family in the LID (residues 89–117) and NMP (residues 41–51) domains, which are involved in binding ATP and AMP, respectively (Figure 1A). The same numbering scheme for the secondary structure elements described for hFap7 has therefore been used (Ren et al. 2005) [11]. Complex formation buries 1,800 Å^2^ of aFap7, representing 18% of the total surface, with interactions over the LID and NMP domains, the C-terminal extension, and residues of the walker B motif (Figure 1A, protein contact map in Figure S2). aRps14 shares 60% sequence identity with yRps14 and can be superimposed on ribosome-bound yRps14 with 1.3 Å r.m.s.d. (Figure 1C, alignment in Figure S1B) [25]. The binding surface on aRps14 is largely comprised of the surface of the β-sheet in the globular core domain (gold in Figure 1A) and the basic residue rich C-terminal extension (Rps14-CE; red in Figure 1A, protein contact map in Figure S2). Comparison of the aFap7–aRps14 complex with ribosome-bound yRps14 did not reveal structural changes in the core domain, with the exception of the β4–α2 loop, whose RNA-bound conformation would clash with aFap7. In contrast, the aRps14-CE is completely remodeled by the interaction with aFap7 (Figure 1C). This large conformational change is possible because of the intrinsically disordered nature of the C-terminal extension. The protein contact map (Figure S2) shows that the interface can be roughly dived into three zones, representing the interaction of the core domains of Fap7 and Rps14, of the Rps14-CE with the aFap7-core, and of the Fap7-CE with the Rps14-core, which suggests that the C-terminal extensions of the two proteins play an important role in the complex.

### The Nonconserved aFap7 Terminus Is Involved in aRps14 Binding

Because of the high sequence identity between the archaeal and yeast proteins, the structure of the aFap7–aRps14 complex presents a good model for the yeast yFap7–yRps14 complex. However, a notable difference concerns the C-terminal extension of aFap7 (dark blue in Figure 1), which is not conserved in yeast and humans in length or sequence. In the aFap7–aRps14 complex, the C-terminal extension folds back onto the globular domain and contains an extra 3–10 helix (η8, dark blue in Figure 1), which packs both into the groove formed by the α5–α6 helices of the LID domain (light blue in Figure 1) on the β-sheet surface of aRps14 (see protein contact map in Figure S2). It contributes 500 Å^2^ of interaction surface with aRps14, including nine hydrogen bonds and one salt bridge. The η8 helix is present in all copies of the complex present in the asymmetric unit, in both ADP and ATP bound forms, and in exactly the same conformation. All these facts seem to indicate that the η8 helix is an integral part of the aFap7–aRps14 complex.

**Figure 2:**
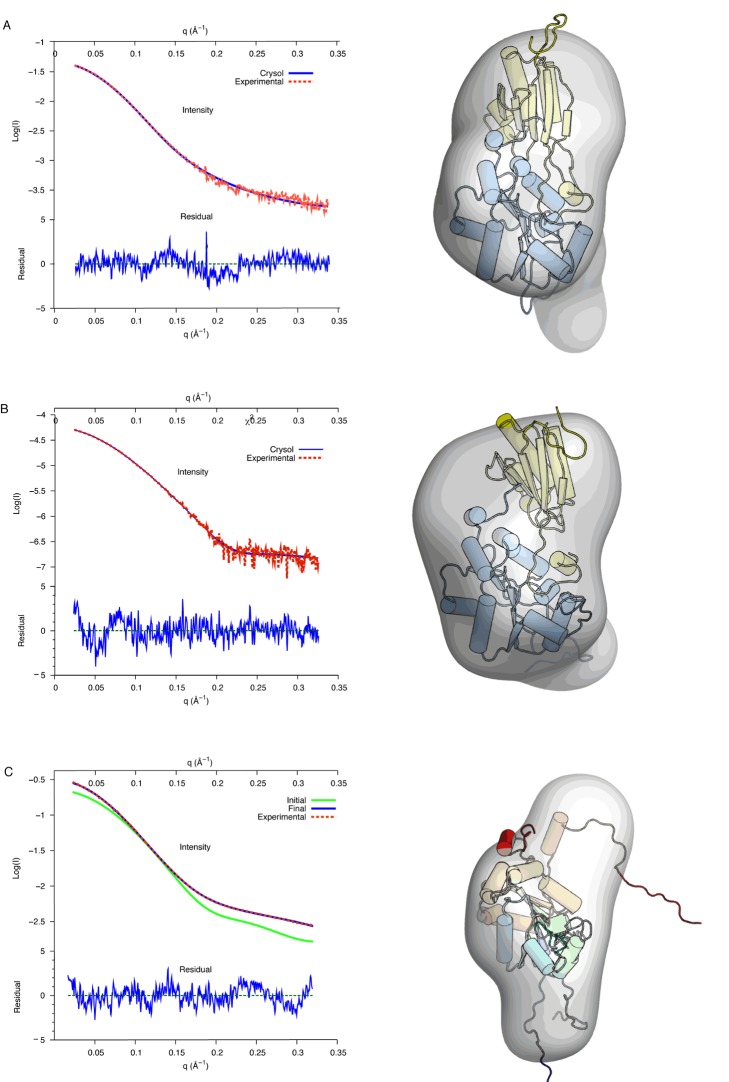
SAXS structure of the yeast and archaeal complexes. SAXS scattering profiles of (A) the aFap7–aRps14 complexes and (B) yeast yFap7–yRps14 complexes and (C) free yFap7. (Left) The experimental scattering profile is depicted in dashed red lines and the calculated profile from the best fit in blue lines. The residual is depicted below the scattering curve. The initial scattering profile of the initial yFap7 model is represented in green. (Right) Representation of the calculated envelope with the best fit model. Rps14 is represented in yellow and Fap7 in blue, except in (C) where yFap7 is colored blue (N terminus) to red (C terminus).

In order to further confirm that the interaction of the η8 helix with Rps14 is not an artifact of crystal packing, we have conducted structural studies in solution by small angle X-ray scattering (SAXS) on the aFap7–aRps14 complex. The resulting SAXS curves were fitted with a model built using our X-ray structure, with flexibility introduced in all missing N-terminal and C-terminal residues and poorly defined loops. The resulting fit shows a good agreement with the crystal structure (Table 2), showing that the η8 helix is located at the aFap7–aRps14 interface in solution (Figure 2A).

**Table 2:** SAXS.

Samples	yFap7	yFap7–yRps14	aFap7–aRps14
Data-collection parameters			
Instrument	SWING	SWING	SWING
Beam geometry (mm)	0.4×0.1	0.4×0.1	0.4×0.1
Wavelength (Å)	1.03	1.03	1.03
q range (Å^−1^)	0.07–0.5	0.07–0.5	0.07–0.5
Exposure time (s)/nb frames	1/100	1/100	1/100
Concentration range (mg ml^−1^)	2–10	5	5
Temperature	288	288	288
Structural parameters			
I(0) (cm^−1^) [from P(r)]	0.21	0.15	0.13
Rg (Å) [from P(r)]	23.35	23.80	24.63
I(0) (cm^−1^) [from Guinier]	0.21	0.15	0.13
Rg (Å) [from Guinier]	23.40	23.80	24.70
D_max_ (Å)	85.5	78.9	85.7
Porod estimate (Å^3^)	49,357	61,505	60,752
Dry volume calculated from sequence (Å^3^)	28,958	46,526	43,889
Molecular-mass determination			
Partial specific volume (cm^3^ g^−1^)	0.710	0.724	0.740
Contrast (Δρ×10^10^ cm^−2^)	3.289	3.032	2.7938
Molecular mass M_r_ [from I(0)]	24,390	37,160	35,421
Calculated monomeric M_r_ from sequence	23,916	38,447	36,272
Software employed			
Primary data reduction	FOXTROT		
Data processing	PRIMUS		
Ab initio analysis	DAMMIF		
Validation and averaging	DAMAVER		
Rigid-body modeling	DADIMODO		
Computation of model intensities	CRYSOL		
Three dimensional graphics representation	PyMOL		

### The Structure of the Fap7–Rps14 Complex Is Conserved from Archaea to Yeast

In order to both confirm the structure in solution of yeast Fap7–Rps14 complex and determine the contribution of the nonconserved C-terminal extension of yFap7, we conducted SAXS studies of yFap7 both free and bound to yRps14. The modeling of yFap7 was performed by introducing flexibility on the N-terminal residues of the tag, the LID domains, the β3–β4 loop, and the entire nonconserved C-terminal extension following the α7 helix, and on the β4–α2 loop in yRps14. The yFap7 terminal extension was modeled in the aFap7 conformation bound to aRps14, stacked on the NMP binding domain. The SAXS data recorded on the free yFap7 clearly indicates that the yFap7 structure contains unstructured regions, as the experimentally determined radius of gyration (Rg) of free yFap7 is comparable to that of the Fap7–Rps14 complex (from both yeast and archaea; Table 2). The modeling of yFap7 with these data clearly shows that a conformation of the C terminus packed on the globular domain is not compatible with the SAXS data (Figure 2C). The most representative structure fits well to the data and shows that the C-terminal extension is unstructured and does not form any contacts with the yFap7 core and NMP domains (Figure 2C). Because the initial conformation was chosen to “favor” the compact conformation during the modeling of the SAXS data, the unwinding of the C-terminal region in order to fit the SAXS data cannot be attributed to an artifact of the modelization protocol.

**Figure 3:**
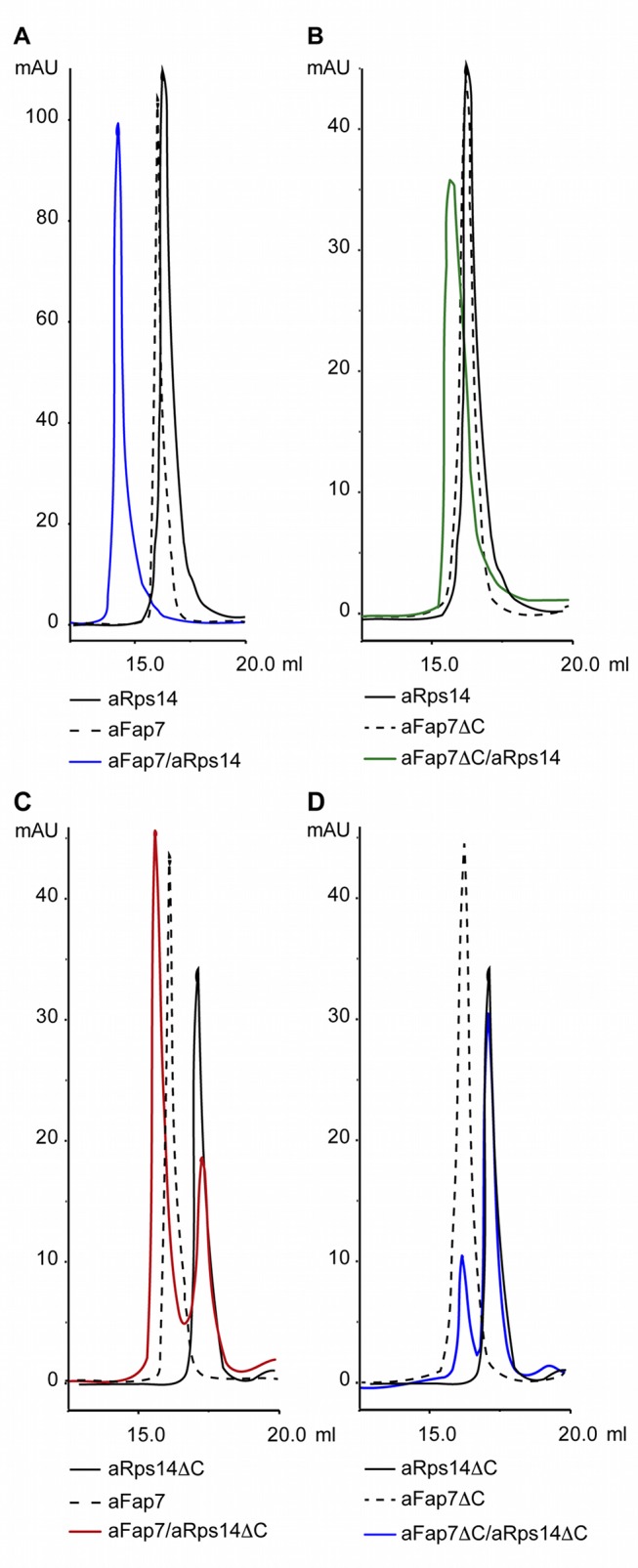
Both the Rps14 and Fap7 C-ter domain participate in their interaction. To analyze Fap7 and Rps14 interaction and the domains involved, their association was observed by analytical gel filtration. Note that the C-terminal deletion of aFap7 removes the only tryptophane, which leads to the reduced absorption in the gel-filtration profile. (A) Gel filtration profiles of aRps14 (black), aFap7 (dotted line), and a 1∶1 mix of aRps14–aFap7 (blue). (B) Gel filtration profiles of aRps14 (black), aFap7ΔC (dotted line), and a 1∶1 mix of aFap7ΔC–aRps14 (green). (C) Gel filtration profiles of aRps14ΔC (black), aFap7 (dotted line), and a 1∶1 mix of aFap7–aRps14ΔC (red). (D) Gel filtration profiles of aRps14ΔC (black), aFap7ΔC (dotted line), and a 1∶1 mix of aFap7ΔC–aRps14ΔC (blue).

Modeling of the structure of the yFap7–yRps14 complex against the SAXS data shows that the complex adopts the same overall structure as its archaeal homologue, with both the same binding surfaces involved and the same orientation between the yFap7 and yRps14 proteins (Figure 2B). The archaeal complex can therefore be used with confidence to model the yeast complex. Surprisingly, the data suggest that the yFap7 C-terminal extension comprising helix η8 adopts the same conformation as the archaeal complex. Firstly, the Rg measured from the SAXS data recorded on the yFap7–yRps14 complex is comparable to that of the archaeal complex, indicating the absence of additional unstructured regions in the yeast complex. In addition, even though the conformation of the C-terminal extension was considered as flexible in the structure calculations, it remains in the same conformation as the aFap7 C-terminal extension in all calculated structures (Figure 2B). This indicates that despite the poor conservation of this region, the yFap7 C terminus participates in yRps14 binding in the same region as the archaeal complex. In agreement with this model, we were not able to purify measurable quantities of yFap7ΔC–yRps14 complex from a polycistronic vector expressing a yFap7 protein truncated of the last 20 residues (unpublished data), although this could be due to insolubility of yFap7ΔC or a poorer expression of yRps14 in this construct. In the archaeal system, truncation of the aFap7 C terminus after helix α7 (residue 157) had no effect on complex formation with aRps14 in our gel filtration assay (Figure 3A,B). However, performing the assay with aRps14ΔC, which shows a reduced but measurable affinity for aFap7 (Figure 3C), leads to a complete dissociation of the complex (Figure 3D). This indicates that the interaction between the Fap7 C terminus and Rps14 extends the binding surface and strengthens the binding affinity of the complex, but is not essential for the formation of the archaeal complex *in vitro*.

### Structure of the Fap7–Rps14 Complex in the ADP and ATP Bound Forms

The structures obtained for aFap7–aRps14 in complex with ADP and ATP show subtle conformational differences. Both nucleotides bind in the aFap7 ATP binding pocket. The ADP-bound state of Rps14-bound aFap7 superposes perfectly with ADP-bound hFap7 for residues of the P-loop and LID domains in the ATP binding pocket (unpublished data). It therefore seems that, in the ADP-bound form, the binding of aRps14 does not lead to major structural changes in the aFap7 LID domain.

**Figure 4:**
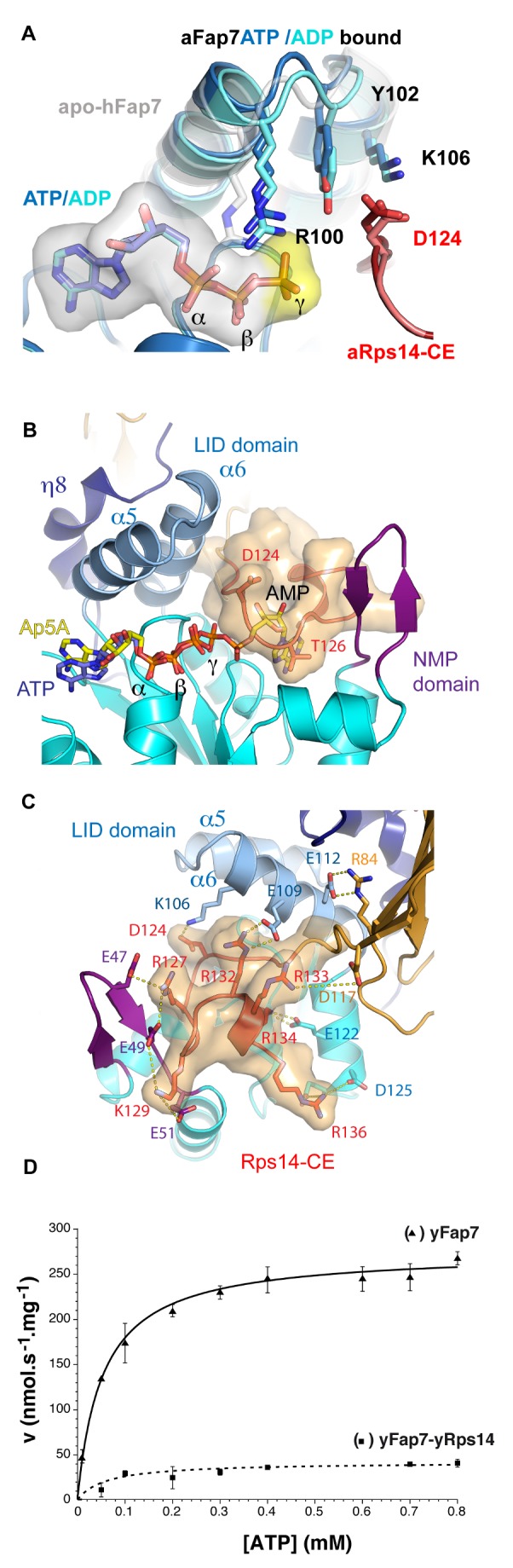
Rps14 obstructs the Fap7 AMP binding cavity. (A) Conformational differences of the ATP binding pocket in complex with ADP (light blue) and ATP (dark blue). The apo-hFap7 structure is represented in grey. (B) Superposition of Ap5A with ATP from AK1 (PDB code 1z83) in the aFap7–aRps14 complex defines the AMP binding pocket. This pocket is filled by the aRps14 C-terminal extension. (C) Cartoon representation of the aFap7–aRps14 complex in the putative AMP substrate binding cavity of aFap7. The C-terminal extension of Rps14 is represented with a transparent surface. Salt bridges to basic residues are indicated. (D) Comparison of AK activities with respect to ATP at a constant concentration of AMP (0.3 mM) of yFap7 (▴) and of the yRps14–yFap7 complex (▪).

In the ATP-bound crystal form, two copies of aFap7 are bound by ATP and two others by ADP. The structures of the ATP- and ADP-bound complexes showed only small, local conformational differences in the actual ATP binding site. The base, ribose, α, and β phosphates of ATP superpose perfectly on those of ADP, and the residues of the P-loop are in the same conformation in both structures (Figure 4A). However, the presence of the γ phosphate of ATP is not compatible with the position of Arg100 (LID domain) in the ADP-bound state (Figure 4A). This arginine, involved in binding the α and β phosphate oxygen atoms in the ADP-bound structure, bridges the α and γ ATP phosphate oxygens. Compared to the ADP-bound structure, the γ phosphate pushes Arg100 away by approximately 1 Å. This effectively distorts the structure of the α5–α6 loop of the LID domain with a 0.9 Å r.m.s.d. compared to the ADP-bound conformation for residues 89–107 (Figure 4A). Interestingly, the conformational change in the LID domain modifies the positions of Tyr102 and Lys106 side chains, which interact with the Rps14-CE Asp124. In the Apo-hFap7 structure, Arg100 would clash with the ADP phosphates, and the LID domain is even more distorted (Figure 4A). This suggests that the LID domain undergoes major conformational rearrangements upon nucleotide binding and/or release and that Arg100 could sense the ATP binding pocket occupation state and relay this signal to the Rps14 binding interface through the LID domain residues Tyr102 and Lys106.

### Rps14 Is a Competitive Inhibitor of Fap7 AK Activity

The AMP binding site on Fap7 homologues has not been experimentally determined, but comparative modeling with related AKs suggests that AMP is bound in the cavity formed by the LID and NMP domains [12]. For illustration, Figure 4B presents the structural model of aFap7 bound to diadenosine pentaphosphate (Ap5A), built by superposing aFap7 with human AK1A bound to Ap5A (Protein Structure Comparison Service Fold at European Bioinformatics Institute, http://www.ebi.ac.uk/msd-srv/ssm, authored by E. Krissinel and K. Henrick). Ap5A is an AK inhibitor that mimics a transition state during phosphate transfer from ATP to AMP. Within Ap5A, one adenosine and the α, β, and γ phosphates superpose perfectly with the bound ATP in the ATP binding site, while the remainder of the molecule matches the AMP substrate binding site between the Walker B, helix α2, LID, and NMP domains.

In the aFap7–Rps14 structure, the C-terminal residues of aRps14 (aRps14-CE) after the final β5 strand forms an unusual interaction with Fap7 that occludes the putative AMP binding site (Figure 4B). The Rps14-CE completely fills the cavity lined by residues from the LID, NMP binding, and Walker B domains, and would preclude nucleotide binding. Specifically, the ribose and base of AMP would sterically clash with aRps14 Gly125 and Thr126 residues (Figure 4B). In the aFap7–aRps14 structure, AMP is therefore excluded from the AMP binding site by aRps14.

The Rps14-CE forms a lasso-like structure, which creates an extensive interaction surface, contributing 800 Å^2^ (45% of the total binding surface) (Figure 4C). In order to confirm the importance of this interaction, we performed protein interaction assays analyzed by gel filtration, using aFap7, aRps14, and an aRps14ΔC construct truncated at residue 117. The aFap7–aRps14 complex could be reconstituted by mixing equimolar amounts of proteins, and this complex migrated as a single peak (Figure 3A). The aFap7–aRps14ΔC sample showed a much lower recovery of the complex, showing that the aFap7–aRps14 interaction was compromised when the aRps14-CE is removed (Figure 3C).

The interaction between the aRps14-CE and the aFap7-NMP domain involves hydrogen bonds between the main chain atoms of aRps14(Arg127/Lys129) and aFap7(Val48/Val50). The aRps14-CE contains six lysine and arginine residues and its interaction with aFap7 involves salt bridges to five conserved basic residues (Figure 4C). Salt bridges are found from acidic aRps14 residues to basic aFap7 residues from the LID domain (Glu109–Arg132), the NMP binding domain (Glu47 and Glu49–Arg127, Glu51–Lys129), and helix α6′ of the CORE domain of aFap7 (Asp122–Arg135, Asp125–Arg136). An intramolecular salt bridge is formed between Arg133 (aRps14-CE) and Asp117 (Rps14 core domain). The only acidic residue in aRps14-CE, Asp124, also forms a salt bridge to Lys106 in the aFap7 LID domain. yRps14–Asp124 does not make any interactions in ribosome-bound yRps14, so its high conservation suggests a major role in Fap7 binding during ribosome biogenesis. All residues involved in these salt bridges are conserved in the yeast and human proteins and found at equivalent positions in the hFap7 structure.

To experimentally verify that Rps14 competes for the AMP binding site of Fap7, we measured the AK activity of free yFap7 and the yFap7–yRps14 complex. As expected, yFap7 has an AK activity with K_m_ and k_cat_ values comparable to those reported for hFap7 (52 µM and 6.12×10^−3^ s^−1^, respectively; Figure 4D). In comparison, the yFap7–yRps14 complex showed almost complete inhibition of the Fap7 AK activity (Figure 4D), with a 94% reduction in enzymatic efficiency (k_cat_/K_m_). This indicates that yRps14 acts as a competitive inhibitor by blocking the AMP binding site and inhibits yFap7 AK activity. In agreement with these results, we found the same AK activity for the aFap7–aRps14ΔC complex and for free aFap7 (Figure S4A). However, because aRps14 ΔC has a lower binding affinity with aFap7, we cannot exclude that this observation of the measured activity arises from loss of interaction with aFap7.

### Fap7 Acts as an RNA Mimic

**Figure 5:**
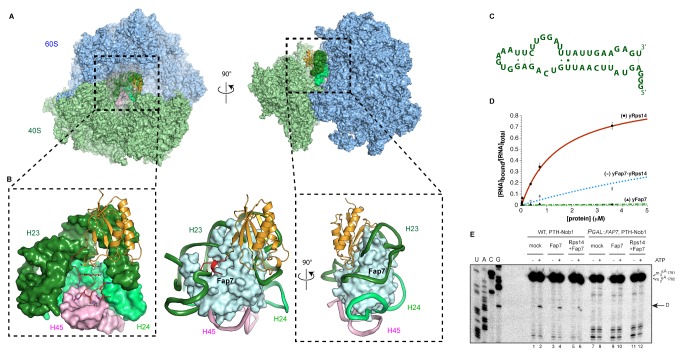
yFap7 acts as an RNA mimic. (A) yRps14 in the yeast ribosome [25]. The 40S is colored green and 60S blue. The Rps26 and Rps1 proteins have been omitted for clarity. Helix 23, 34, and 45 are colored light green, dark green, and pink, respectively. (B) Close-up view of the yRps14 interaction with RNA. The Fap7 protein placed in the same position as in the aFap7–aRps14 complex is overlaid in blue surface representation (middle and right). (C) Secondary structure of helix 23 from *S. cerevisiae* 18S rRNA (nucleotides 884 to 928 with GGG extension at 5′ end) used for binding assays. (D) Quantification of bound RNA ratio versus total RNA in each well and nonlinear curve fitting of the data points. Interaction of RNA with yRps14 (•), yRps14–yFap7 complex (▪), and yFap7 (▴). (E) Primer extension analysis showing the activation of *in vitro* site-D cleavage in pre-40S particles purified, using PTH-NOB1 from either wild-type cells (WT) or after depletion of Fap7 (PGAL::FAP7), by nucleotide addition. The strong upper stops result from termination at the sites of 18S rRNA base-methylation at A1779 and A1780. These modifications precede site-D cleavage *in vivo*. The arrow indicates site D. ATP was added at 1 mM when indicated. We added 250 pmoles of recombinant Fap7-his or Fap7–Rps14 complex when indicated.

Because Rps14 is a structural constituent of the ribosome, we compared the structure of yRps14 in complex with the ribosome or in complex with Fap7. In the context of the ribosome, yRps14 is positioned in the platform domain and buries 2,000 Å^2^ upon binding 18S RNA (Figure 5A). yRps14 binds the stem of helix 23 (H23) of 18S rRNA through the surface of the β-sheet on the β1 to β4 strands, the β1–β2, β2–β3, and β4–α2 loops, whereas the loop of rRNA helix 24 (H24) is bound by the β3–α1 loop (Figure 5B). In the ribosome, the Rps14-CE is totally buried and involved in extensive protein–RNA interactions, as frequently found for the charged C-terminal extensions of ribosomal proteins [26]. The six conserved, basic residues in the Rps14-CE (out of seven total) form salt bridges to phosphates of helix 24 and helix 45 (H24 and H45 in Figure 5B).

Surprisingly, the surface of Rps14 involved in rRNA binding corresponds closely to the interaction surface with Fap7 in the aFap7–aRps14 complex (Figure S3). The Rps14 interface involves 45 interfacing residues in the aFap7–aRps14 complex (green in Figure S3, left) and 50 in the complex with the 18S rRNA (purple in Figure S3, right). Comparison of these residues shows that 35 residues (over 70% of the total) are common between the two interfaces. All of the Rps14 structural elements involved in RNA binding are also involved in protein interactions, except for residues of the β3–α1 loop.

Closer inspection revealed that the specific protein–RNA contacts formed by Rps14 in the ribosome are replaced by contacts to protein side chains in the aFap7–aRps14 complex. For example, in ribosomes the globular domain of yRps14 binds the 18S rRNA via hydrogen bonding with Asn24 and salt bridges with Arg41, Arg84, and Lys90, whereas in the aFap7–aRps14 complex these residues contact aFap7–Asp117, Asp169, Glu112, and Asp125, respectively. This correspondence is even more dramatic for the Rps14-CE, where all six salt bridges to RNA are replaced by salt bridges involving protein side chains (Figures 4C and 5B). Fap7 therefore acts as an RNA mimic, which achieves specific protein–protein contacts by mimicking protein–RNA contacts.

To experimentally verify that Fap7 competes with RNA for Rps14 binding, we performed filter binding assays using a radioactively labeled 45 nt RNA oligomer corresponding to Helix 23 of the 18S rRNA from *S. cerevisiae* (residues 884–928, Figure 5C and Figure S4C,D,E,F,G) to mimic the binding site of Rps14. MBP-yRps14 was competent in binding this construct with an apparent affinity of 1.50±0.15 µM (Figure 5D, red), whereas yFap7 did not show detectable binding (Figure 5D, green). The yFap7–yRps14 complex was severely impaired in RNA binding (Figure 5D, blue), with a measured affinity of 14.8±4.5 µM. This confirms that *in vitro* Fap7 binding to Rps14 effectively occludes the RNA binding interface and can prevent Rps14 from binding a synthetic RNA construct. The consequence of this finding is that during ribosome maturation the structure of the platform domain must undergo a large change in protein and/or rRNA conformation to accommodate formation of the Fap7–Rps14 complex.

### In Vitro Maturation of Preribosomes Purified in the Absence of Fap7

Depletion of yFap7 leads to accumulation of pre-80S–like complexes, composed of pre-40S particles associated with mature 60S subunits, but three components of the platform domain—Rps14, Rps26, and Rps1—are underrepresented in these particles [5]. To assess whether pre-80S complexes purified from yFap7-depleted cells represent ribosome assembly intermediates, we used an *in vitro* maturation assay to monitor 20S pre-rRNA cleavage by the yNob1 nuclease at site D [4]. In preribosomes purified from wild-type yeast using PTH–Nob1 as bait, cleavage was activated by addition of ATP (Figure 5E and quantifications in Figure S4I), as previously reported [4]. In contrast, preribosomes (in 80S-like Fap7-depleted conformation) purified by PTH–Nob1 in Fap7-depleted cells are not competent for site D cleavage (Figure 5E). These data confirm that preribosomes assembled in the absence of yFap7 are not substrates for the Nob1 nuclease probably due to incomplete assembly in the platform domain.

Fap7 depletion results in loss of Rps14 from pre-80S particles, suggesting that reintroduction of Rps14 might rescue site D cleavage *in vitro*. We reasoned that Fap7 could act as an assembly factor to reincorporate Rps14 in the ribosome. However, addition of yFap7 or yFap7–yRps14 in the absence or the presence of ATP/Mg did not lead to any activation of D-site cleavage, in wild-type preribosomes or preribosomes purified from Fap7-depleted cells. These results show that Fap7–Rps14 cannot remodel the complex into an active conformation. Mass spectroscopy analysis of the purified particles shows the preribosomes purified by PTH–Nob1 in wild-type and Fap7-depleted cells both contain the 40S and 60S ribosomal proteins in roughly the same proportions, but assembly factors are overrepresented in wild-type preribosomes (Figure S4H and Table S1). The lack of cleavage could therefore arise because factors required for Fap7 activity *in vivo* are absent in the *in vitro* assay, because the experimental conditions are not optimal for the activity of the Fap7–Rps14 complex or because the pre-80S complexes generated in the absence of Fap7 correspond to dead-end intermediates.

In the preribosomes purified in the absence of Fap7, we observed rRNA degradation bands, consistent with an altered structure of the 20S rRNA within the pre-40S particles (Figure 5E). Several rRNA structure-probing studies indicate conformational plasticity of the 3′ region of the 18S rRNA during ribosome biogenesis [7],[26],[27]. In the absence of yFap7 or in the Rps14–R134A mutant background, an 18S rRNA degradation product (referred to as 17S RNA) missing Helices 45 and 44 is observed [14],[19]. This indicates that compromising the function of Fap7–Rps14 causes the 20S pre-rRNA to adopt a nonnative conformation that is prone to degradation. This is in line with electron microscopy structures of the pre-40S particles, which show that the platform domain is assembled even though Rps14 is not at its final position [8]. Further experiments are needed in order to determine if Fap7–Rps14 has to be correctly incorporated prior to formation of pre-80S particles or if Fap7 is present in the pre-80S particles during the final translation-like steps that confer competence for site-D cleavage.

### Function of Fap7 Catalytic Activity in Ribosome Biogenesis

The binding interface of the aFap7–aRps14 complex involves residues important for both the catalytic activity of Fap7 and the RNA binding capacity of Rps14. The double D82A–H84A mutation in yFap7, located in the Walker B motif, was shown to inhibit 20S processing in the same way as *FAP7* deletion [14], and the corresponding mutation in hFap7 (H79G) abolishes ATPase and AK activities [12]. This suggested that nucleotide binding and/or hydrolysis by Fap7 might regulate the binding of Rps14 to the rRNA. To test this hypothesis, we evaluated the roles of nucleotides in formation or dissociation of the Fap7–Rps14 complex.

**Figure 6:**
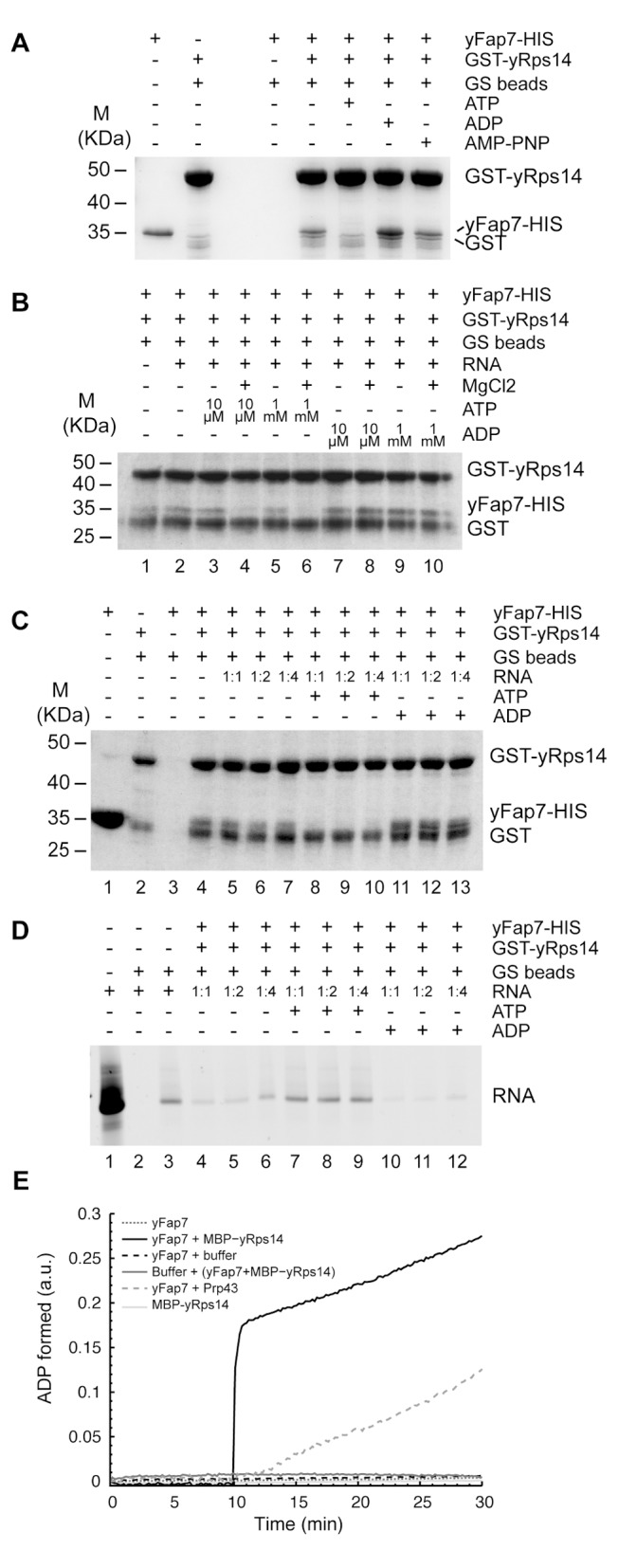
Fap7 ATPase activity regulates its association with Rsp14. Interaction between yFap7 and GST-Rps14 was tested by pulldown experiments. (A) Interaction of yFap7 with GST-Rps14 was tested by addition of 800 pmoles of yFap7 on GST-yRps14 beads resuspended in 1 ml of IP buffer. Effect of addition of ATP, ADP, or AMP-PNP at 1 mM final concentration in the presence of MgCl2 (5 mM) was tested. Protein associated with the beads was analyzed by Coomassie staining. For input controls, 10% of Fap7 (80 pmoles) and the same quantity of Rps14 beads used for the IP were loaded. (B) Effects of magnesium and nucleotide concentration were tested by using the same strategy in the presence of RNA (cf., D). Two quantities of nucleotides were used: 1 µmole (1 mM) or 10 nmole (10 µM). Experiments were done in the presence or in the absence of 5 mM MgCl_2_. (C) Association of GST-yRps14 to RNA was assessed by a competition experiment using a different ratio between RNA and yFap7: 800 pmole Fap7 with 800 pmole RNA (1∶1), 400 pmole Fap7 with 800 pmole RNA (1∶2), and 200 pmole Fap7 with 800 pmole RNA (1∶4). Nucleotides were added at 1 mM final. Protein associated with the beads was analyzed by Coomassie staining. For input controls, 10% of Fap7 (80 pmoles) and the same quantity of Rps14 beads used for the IP were loaded. (D) Same as in C, but this time the RNA counterpart was followed on Urea-acrylamide gel after SYBR Safe staining. For input control, 80 pmole of RNA was loaded. (E) ATPase activity of yFap7 (black points) was followed by a coupled enzyme assay. Effect of addition after 10 min of MBP-yRps14 (black line), buffer (dotted dark line), or Prp43 (dotted dark grey line) was also monitored. In parallel, ATPase activity of MBP-yRps14 (light grey line) alone and the preformed complex yFap7–yRps14 (dark grey line) was also tested.

To determine whether nucleotide binding strengthened or weakened the yFap7–yRps14 interaction, we used a pull-down assay with GST-tagged yRps14 bound to glutathione resin. The beads were incubated with yFap7 and/or RNA (H23 helix construct, Figure 5C) in the presence of various combinations of nucleotides and magnesium, the beads were washed with 30 volumes of buffer, and the bound proteins were revealed by SDS-PAGE. In the same conditions and where appropriate, bound RNA was precipitated and revealed on an acrylamide-urea gel. We find that in the absence of nucleotides or in the presence of ADP+Mg, yFap7 is able to bind yRps14 (Figure 6A, lanes 4 and 6). However, in the presence of ATP+Mg, almost no yFap7 was retained by yRps14, indicating that ATP+Mg severely reduces the affinity of yFap7 for yRps14 (Figure 6A, lane 5). In order to determine if this effect was due to ATP binding in the yFap7 ATP binding site or to ATP hydrolysis, we performed this experiment in the presence of the nonhydrolysable analogue AMPPNP (Figure 6A, lane 7). AMPPNP had no effect on yFap7 binding, indicating that hydrolysis and not binding is required for complex dissociation. This was further confirmed by performing the assay in the absence or presence of magnesium. The yFap7–yRps14 complex was dissociated (or failed to form) only in the presence of ATP+Mg (lanes 4 and 6), further confirming that ATP hydrolysis modulates the protein–protein interaction. To ensure that was not due to loss of nucleotide binding, we have verified by native gel electrophoresis using radiolabeled nucleotides that Fap7 binds AMPPNP and ATP−Mg with the same (AMPPNP) or a slightly lower (ATP−Mg) affinity compared to ATP+Mg (Figure S4B). No difference in the effect was observed at high (1 mM) or low (10 µM) nucleotide concentrations (Figure 6B), indicating that the effect was also not due to competition for the yRps14 binding site. Interestingly, no effect of ATP+Mg was observed when the assay was performed solely at 4°C. Incubation of the mix at room temperature is absolutely necessary, consistent with the model that the catalytic activity of the enzyme is responsible for the effect (unpublished data). This effect also explains why this effect was not seen in experiments performed on hyperthermophylic archaeal proteins at low temperatures [19].

We have monitored the stimulation of ATPase activity in our coupled enzyme assay (Figure 6E). The ATPase activity of yFap7 (5 µM) as a function of time was comparable to the background levels without yFap7. After 10 min incubation, Prp43 (a helicase harboring ATPase activity) or MBP-yRps14 were injected at the same concentration as yFap7. For Prp43, we observed an increased rate of production of ADP, reflecting the ATPase activity of the protein. Similarly, addition of yRps14 resulted in a rapid burst of ATP hydrolysis, followed by a linear increase in ADP formation, demonstrating that ATP hydrolysis by Fap7 is stimulated by yRps14 (Figure 6E). In contrast, addition of the preformed yFap7–yRps14 complex to the ATP buffer did not lead to a measurable increase in ATPase activity. This is in line with the observation that dissociation of the yFap7–yRps14 complex in the presence of ATP+Mg could only be observed by mixing the separately purified proteins, and not with the yFap7–yRps14 complex purified by co-expression (Figure S5). This explains why the co-purified complex, already in a stable conformation, could be crystallized in the presence of ATP+Mg and possibly shows that an external factor is necessary to trigger ATPase activity and dissociate the fully assembled complex *in vivo*.

In order to determine whether the nucleotide-dependent binding of Fap7 to Rps14 modulates the binding of Rps14 to RNA, this assay was repeated with GST-Rps14 that had been preincubated with an increasing ratio of RNA (Figure 6C and D). In agreement with the filter binding assays, yRps14 efficiently remained associated with the RNA construct in the absence of Fap7, and yFap7 was capable of displacing RNA from yRps14. yFap7+ADP was more effective in displacing RNA than yFap7 in the absence of nucleotides (compare lanes 5–7 and 11–13), while ATP+Mg failed to compete with RNA (lanes 8–10). This indicates that ADP and ATP modulate the affinity of the Fap7–Rps14 complex and could indirectly regulate the binding of Rps14 to RNA through this interaction.

### Model of Fap7 Catalytic Activity in Complex Dissociation

Two mechanisms by which hydrolysis could dissociate the yFap7–yRps14 complex can be envisaged. The first explanation is that ATP hydrolysis induces a “static” modification of the structure (or of the oligomerisation state) of yFap7 and/or yRps14 that renders it incapable of forming the complex. One such modification compatible with the biochemical and structural results would be that Fap7 acts as a protein kinase to phosphorylate Rps14. The location of the Rps14-CE in the binding site for the AMP substrate of aFap7 would be consistent with this model. This mechanism would potentially be similar to the phosphorylation-dependent incorporation of Rps3 into pre-40S by the Hrr25 protein kinase [28],[29]. The γ phosphate of ATP is positioned at 5 Å from the Cα atoms of aRps14–Asp124, comparable to the distance to the AMP substrate. Moreover, a serine is found in the position equivalent to Gly125 in humans and yeast, and has been identified as phosphorylated in high-throughput assays [30]. The ability of yFap7 to phosphorylate yRps14 was assessed by incubating yFap7 and the yFap7–yRps14 complex with [γ-^32^P] ATP, but no evidence of phosphorylation of yRps14 or autophosphorylation of yFap7 was detected (unpublished data). However, phosphoaspartate regulation of Rio2 assembly has already been demonstrated [10], and because phosphoaspartates are very unstable, the phosphorylation of Asp124 warrants further investigation. This would also be consistent with the burst of ATPase activity seen in our assay upon addition of Rps14.

An alternative model would be a “dynamic” mechanism, in which complex dissociation is driven by conformational changes powered by ATP hydrolysis. This is potentially similar to the large-scale conformational changes in the ATP and AMP binding site observed upon substrate binding and nucleotide hydrolysis in AKs. This involves the transition between a closed (ATP- and AMP-bound) and an open (apo) conformation of the LID and NMP domains [31]–[33]. This transition is coupled to local unfolding (cracking) that results from the release, during catalysis, of intramolecular strain built up by residues in the LID domain [34],[35]. In Fap7, such a local unfolding event of the LID domain during catalysis is strongly predicted to disrupt the Rps14 binding interface. Conversely, nucleotide binding in the ATP binding pocket would stabilize the closed conformation and by extension the Fap7–Rps14 interaction surface. This model is consistent with the observation that ADP binding increases the affinity of yFap7 for yRps14 and that ATP hydrolysis destabilizes the complex.

**Figure 7:**
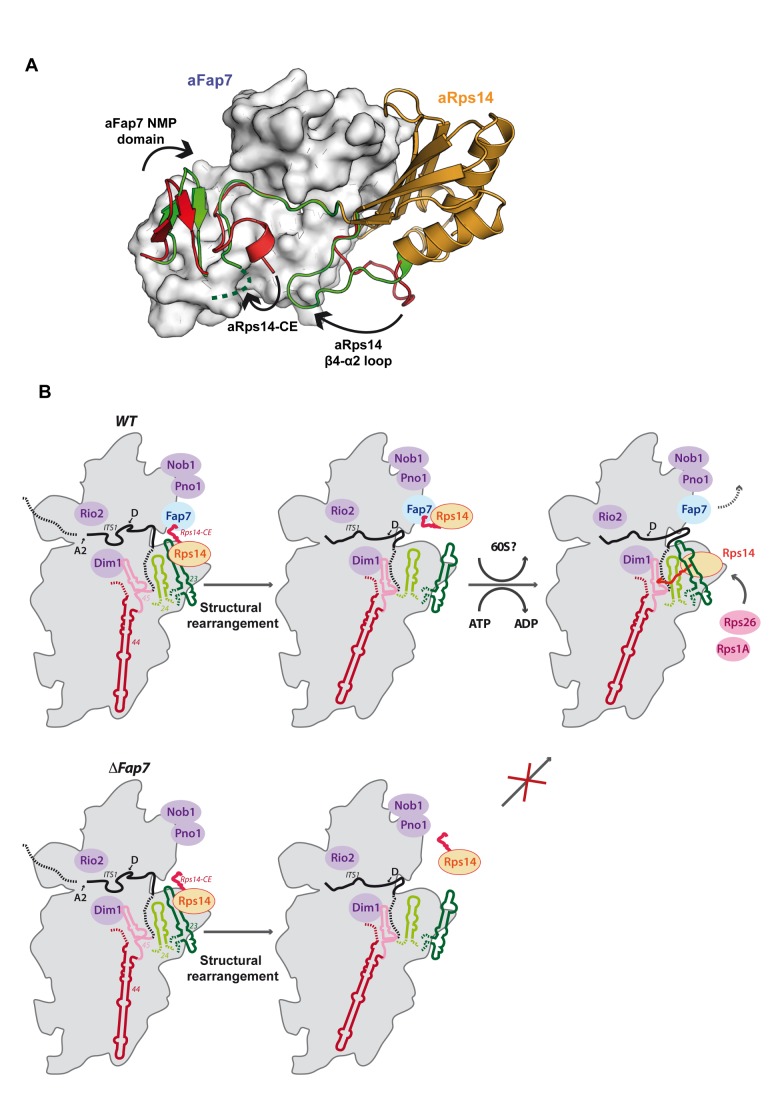
Model of Fap7 function in ribosome biogenesis. (A) Model of the conformational change leading to Rps14 release. The two conformations of the aFap7 NMP domain, the Rps14-CE and Rps14 β4-α2 loop, are represented in red and green. The remaining aFap7 is in surface representation. The seven disordered C-terminal residues of the green conformation are depicted by a dashed line. The extrapolated movement of the three regions suggested to accompany disruption of the Fap7–Rps14 complex is indicated by black arrows (see also Movie S1). (B) During ribosome maturation, Fap7 and Rps14 are bound in the platform domain, which is in a nonnative conformation. After A2 cleavage, Fap7 removes Rps14 from the rRNA. Rps14 is then dissociated from Fap7, probably following ATP hydrolysis, and reincorporated in a near cognate position. This enables a major conformational change to occur that restructures the platform domain and the ITS1, delivering a pre-40S that can undergo the final cleavage at site D. In the absence of Fap7, Rps14 cannot be reincorporated in the ribosome, the rRNA in the platform domain is not remodeled, and cleavage of the D site is not possible, leading to a dead end in the pathway.

A specific model for the conformational change can be proposed from the different structures of the aFap7–aRps14 complex. In the four copies of the complex present in the asymmetric unit in each space group, a second conformation of the aFap7–aRps14 complex is observed, which differs in the structure of three regions: the LID domain of aFap7, and the C-terminal extension and β4–α2 loop of aRps14. This structure represents a more open conformation of the NMP domain, a partially unstructured Rps14-CE (loss of four salt bridges) and a conformation of the aRps14 β4–α2 loop that would clash with a structured Rps14-CE (Figure 7A). We propose that the different conformations observed for the aFap7–aRps14 complex reflect changes relevant to Fap7 function and suggest a series of concerted motions that favor Rps14 disassembly: (1) Hydrolysis of ATP unfolds the LID domain helices, which affects interactions with both the Rps14-CE and the core Rps14 domain; (2) the Rps14-CE is destabilized and the salt bridges are broken; (3) the NMP domain β-hairpin shifts to an open conformation favoring the release of the aRps14-CE; and (4) the Rps14 β4–α2 loop “pushes” the Rps14-CE out of the substrate binding cavity (Figure 7A and Movie S1).

## Discussion

### Fap7 Chaperones Rps14 Incorporation by RNA Mimicry

The Fap7–Rps14 complex is the first structure of a ribosome assembly factor bound to a ribosomal protein. The structure of the complex is very informative because it shows that the binding interface involves regions important both for the catalytic function of Fap7 and for RNA binding by Rps14. Fap7 appears to sequester Rps14 by blocking its RNA binding site in an ATP/ADP-dependent mechanism. Binding of Fap7 with Rps14 is not compatible with the position of Rps14 in the mature ribosome, and preribosomes purified in the absence of Fap7 do not contain stoichiometric amounts of Rps14. This suggests that Fap7 is involved in incorporating and/or repositioning Rps14 in the pre-40S particles. Fap7 would therefore function as a chaperone, enabling the final assembly of Rps14 in the ribosome at a particular stage in the maturation pathway. A model for this is presented in Figure 7B.

The remarkable correspondence between the RNA and Fap7 binding interfaces of Rps14 indicates that Fap7 chaperones the assembly of Rps14 by mimicking RNA. Interactions of Rps14 with protein side chains in the Fap7–Rps14 complex are replaced by RNA interactions in the mature ribosomal subunit. RNA mimicry is relatively rare, and only a few proteins are known to show this property in RNP assembly pathways [36],[37]. The Fap7–Rps14 complex is also notable because the binding of Rps14 inhibits the adenyl kinase catalytic function of Fap7. Inhibition of nucleases by proteins with RNA mimic function have already been observed for the immunity proteins of colicin nucleases [38], but Fap7 is unusual because the RNA mimic is the enzyme that is inhibited.

Although Fap7 harbors AK activity, the structure of the Fap7–Rps14 complex suggests that this enzymatic activity is not used directly in ribosome biogenesis. We speculate that in a common ancestor to Archaea and Eukaryotes, Fap7 had an AK-related function in the nucleus but was reprogrammed by gaining the capacity to bind Rps14, thus enabling Fap7 to regulate ribosome biogenesis. The biological relevance of nuclear AK activity has not been demonstrated directly, but AMP/ATP signaling networks are important in the nucleus and ribosome biogenesis is an energy-consuming process. Under stress conditions, such as nutrient deprivation, rapid inhibition of ribosome synthesis may be important to conserve energy. The AMP-activated protein kinase (AMPK) monitors nutrient availability and cellular energy status, through the AMP/ATP ratio, and regulates rRNA transcription, but is otherwise unrelated to Fap7 [39],[40]. It has also recently been proposed that AMPK responds to ADP/ATP ratios [40]–[42]. Moreover, nuclear transport can also be regulated by AK activity [43]. Because Rps14 and AMP could compete for the same binding site on Fap7, we propose that the *in vivo* function of Fap7, and therefore maturation of 40S ribosomal subunits, would be inhibited at elevated levels of AMP, which might signal reduced energy availability. A deregulation of the ADP/ATP ratios would also interfere with the Fap7–Rps14 association and/or dissociation and affect ribosome biogenesis. The reported role of Fap7 in oxidative stress responses [44] might also be mediated by the effects deregulated ADP/ATP ratios on Fap7–Rps14 association and dissociation. Notably, the inhibition of the late, cytoplasmic steps in 40S subunit synthesis in yeast leads to accumulation of pre-40S particles, which might therefore remain available for subsequent processing. This is in contrast to the inhibition of earlier, nuclear steps in ribosome synthesis, which leads to rapid degradation by RNA surveillance systems.

### A Major Conformation Rearrangement of the Platform Domain

Rps14 is an essential structural component of the ribosome and, together with Rps5, is involved in helping to form or maintain the structure of the head/platform domain [45]. However, although mutations in the C-terminal extension of Rps14 interfere with site-D cleavage [20], depletion of Rps14 leads to earlier defects in pre-rRNA processing [46]–[48]. Rps14 therefore has a dual role in ribosome biogenesis, acting in both early and late preribosomal particles, as is the case for Rps5 [45]. This indicates that Rps14 is bound to the ribosome throughout the maturation process, so its function in 20S processing must be triggered by an external factor or conformational change. Fap7 is localized in both the nucleus and cytoplasm, suggesting that it too is bound early in preribosome maturation, although the timing of Fap7–Rps14 complex formation is unclear. A major implication of the structure of the aFap7–aRps14 complex is that Fap7 binding is not compatible with the structure of ribosome-bound Rps14. Thus, binding of Fap7 to Rps14 would prevent the final 40S subunit association of Rps14. Rps14, Rps1, and Rps26 are underrepresented in late preribosomes in the absence of Fap7 [5]. This argues that Fap7 does not simply block binding of these proteins, but is required for their correct assembly.

EM structures of preribosomes also show that the platform is not completely assembled: Rps14 is not in its final position, Pno1–Dim2 overlaps with the Rps16 binding site, and the rRNA, notably Helix 44 and the decoding site, is shifted from its position compared to the mature ribosome structure [8]. In addition, the presence of the 212 nt ITS1 region, on the 3′ end of the 18S rRNA, potentially creates steric hindrance in pre-40S particles and may distort the structure of the platform domain. A major conformational change may therefore be necessary to reposition the Nob1 at site D, and this was suggested to occur during the translation-like cycle in the pre-80S ribosomes [4]. The pre-40S EM structures suggest that Rps14 is already present in the platform domain before cleavage, and we speculate that prior to the conformational rearrangement, Fap7 bound to Rps14 functions to prevent premature assembly of the platform.

### Function of Fap7 in Ribosome Biogenesis

Our results suggest a model for the function of Fap7 in ribosome biogenesis (Figure 7B). We propose that in early pre-40S ribosomes prior to conformational reorganization of the platform domain, Rps14 is located in the platform domain but has not adopted its binding site in the mature ribosome. Prior to or during the structural rearrangement of the platform, Fap7 transiently binds Rps14, displacing bound RNA. Subsequent dissociation of the Fap7–Rps14 complex by ATP hydrolysis allows Rps14 to rebind the 18S rRNA, which may assist its folding into the mature conformation. After this step, the pre-40S subunits are ready for association with Fun12 and mature 60S subunits and can undergo the final site-D cleavage in pre-80S particles. Although our model is consistent with the experimental results for the *Pyrococcus horikoshii* archaeon [19], the model we propose is different because of the additional information provided by the structure of the complex and because biochemical assays, performed on the yeast proteins at physiological temperatures, uncovered a link between ATP hydrolysis and complex dissociation.

It remains to be determined how Fap7 and the Fap7–Rps14 complex associate with the ribosome. Because the Rps14 C terminus is specifically required for the late assembly steps, it is possible that Rps14 binds both rRNA and Fap7 through the Rps14-core domain and C-terminal extension, respectively. It is also possible that Fap7 is additionally anchored in the preribosomes by interaction with other proteins. Consistent with this, protein cross-linking identified Fap7 contacts with several proteins in the preribosomes [5]. An interaction between aFap7 and aNob1 has also been observed [19], but we have not been able to observe this interaction with the yeast homologues.

The model that Rps14 is initially bound in the vicinity of its final location but then repositioned by Fap7 is conceptually related to the assembly of Rps3 [29]. Rps3 is initially weakly associated with preribosomes but is dissociated by phosphorylation by the Hrr25 protein kinase. After the subsequent formation of the beak structure in the pre40S, dephosphorylated Rps3 reintegrates the ribosome in its final position. This assembly pathway is akin to the assembly of Rps14 by the Fap7 kinase, which is linked to a structural rearrangement in the platform domain. Given the complexity of preribosome assembly and the huge numbers of factors involved, each of these two-step assembly pathways may enhance overall speed and efficiency. Several other proteins have been reported to act as “place holders” that occupy ribosomal protein binding sites prior to recruitment of the ribosomal protein itself into the maturing structure [49],[50]. It has also been proposed that dispersed pre-rRNA regions can initially be brought together by ribosome synthesis factors, prior to binding by ribosomal proteins that will lock these regions into their final positions in the mature ribosome [51]. It therefore appears that ribosome assembly not only proceeds sequentially by addition of factors but also involves major rearrangements in ribosomal protein binding sites that have yet to be fully characterized.

Finally, the structure of the Fap7–Rps14 complex provides a molecular framework for the design of inhibitors of the interaction. These might serve to reactivate p53 expression in cancerous cells in which ribosome biogenesis has been deregulated. Our structure suggests that molecules targeted to the Rps14-CE binding cavity could effectively interfere with ribosome biogenesis in a similar manner to Rps14-CE mutations. The structure of the cavity offers the potential for designing much more selective inhibitors than those targeted to the ubiquitous ATP binding pocket. Disruption of the Fap7–Rps14 interaction should induce cell cycle arrest by both deregulating ribosome biogenesis and perturbing the Rps14–MDMd2–p53 pathway. This combination of p53-dependent and -independent effects could potential provide powerful chemotherapeutic approaches [52].

## Materials and Methods

### Cloning, Expression, and Purification

The open reading frames of both genes of Fap7 and Rps14 from *S. cerevisiae* and from *P. abyssi* were synthesized commercially by Genscript Corp (Piscatawy, NJ) and inserted as polycistronic constructs in pET21(a+) (Novagen). The pET21–Fap7–Rps14 constructs contain an N-terminal 6xHis-tagged Fap7 fusion protein. The constructs were expressed in Rosetta 2DE3 strain from *Escherichia coli* (Invitrogen) at 37°C in LB medium (Sigma) supplemented with ampicillin (100 µg ml^−1^) and chloramphenicol (25 µg ml^−1^) until OD600 nm between 0.6 and 0.8. Recombinant protein expression was induced by addition of 1 mM isopropyl β-D-1-thiogalactopyranoside. Cell cultures were incubated 4 h at 37°C and then harvested by centrifugation and resuspended in buffer A (20 mM Tris-HCl pH 8, 300 mM NaCl, 40 mM Imidazole) supplemented with 1 mM phenylmethylsulfonyl fluoride. Cells were lysed by using a French Press (4 runs at 11,000 p.s.i.), and lysate was centrifuged for 30 min at 20,000 rpm. The clear lysate was loaded onto a 5 ml HisTrap (GE Healthcare) connected to an ÄKTA purifier (GE Healthcare). Nonspecific proteins were removed by washing the column with buffer A, and the Fap7–Rps14 His-tagged complexes were then eluted with a linear gradient of imidazol (buffer B, 20 mM Tris-HCl pH 8, 300 mM NaCl, 500 mM Imidazole). Gel filtration was then performed on the fractions containing eluted proteins using buffer C [20 mM Tris-HCl pH 8, 200 mM NaCl, 1 mM Dithiothreitol (DTT)] on a Superdex 75 26/60 (GE Healthcare).

For GST-yRps14 and MBP-yRps14, the open reading frame of Rps14 from *S. cerevisiae* was cloned into pETM30 and pETM41, respectively (courtesy of A. Geerlof, EMBL Hamburg Outstation), and for His-yFap7, the open reading frame of Fap7 from *S. cerevisiae* was cloned into pRSF-DUET (from S. Granneman). For all the archaeal proteins, aRps14, aFap7, aRps14-ΔC, and aFap7ΔC, the open reading frames from *P. abyssi* were synthesized commercially by Genscript Corp (Piscatawy, NJ) and inserted in pETM11 (courtesy of A. Geerlof, EMBL Hamburg Outstation). All these constructs were expressed in Rosetta 2DE3 strain from *E. coli* (Invitrogen). Cells were grown in LB at 37°C to an OD of 0.5, and then protein expression was induced with 0.5 mM IPTG for 4 h at 37°C for archaeal proteins and 16 h at 18°C for yeast proteins. Cells were lysed by sonication, and the lysate was centrifuged for 30 min at 20,000 rpm.

For protein purification, the clear lysate was loaded onto a 5 ml HisTrap (GE Healthcare) connected to an ÄKTA purifier (GE Healthcare) for His-tagged proteins. Nonspecific proteins were removed by washing the column with buffer A′ (50 mM Hepes pH 7.5, 300 mM KCl, and 40 mM Imidazole), and the His-tagged complexes were then eluted with a linear gradient of imidazol (Buffer B′, 50 mM Hepes pH 7.5, 300 mM KCl, 500 mM Imidazole). Gel filtration was then performed on the fractions containing eluted proteins using buffer C′ (50 mM Hepes pH 7.5, 300 mM KCl, 1 mM DTT) on a Superdex 75 16/60 (GE Healthcare).

For IP assays, we prepared beads with GST-yRps14. Cells were harvested by centrifugation, and pellets were resuspended in 5–8 volumes of IP buffer (300 mM KCl, 50 mM Hepes pH 7.5, 0.5 mM EDTA, 1 mM DTT, 10% Glycerol) containing protease inhibitor complete EDTA free from Roche. Cells were broken by sonication, and the extract was clarified by centrifugation at 20,000 rpm for 30 min. Clarified extract was then incubated with pre-equilibrated glutathione sepharose beads from GE at a ratio of 0.5 ml of bead for each litter of extract, overnight at 4°C. Beads were then washed three times with 10 volumes of IP buffer and stored at 4°C.

### Crystallization, Data Collection, and Processing

The crystallization trials were performed using the hanging-drop vapor diffusion technique in 1 µl drops (with a 1∶1 protein∶precipitant ratio) equilibrated against 500 µl reservoir solution at 18°C. The two different crystal forms were obtained in 0.2 M MgCl2, 0.1 M Tris-HCl pH 8.5, 25% (w/v) polyethylene glycol 3350 with the aFap7–aRps14 complex at 3 mg ml^−1^ supplemented with 1 mM MgCl_2_ and either 1 mM ADP or 1 mM ATP.

Crystals were cryoprotected using three successive soaking steps with the reservoir solution containing 10%, 20%, and 30% ethylene glycol. X-ray data were collected at the Soleil Synchrotron (Saint-Aubin, France) on Beamline Proxima1 and at ESRF (Grenoble, France) on ID23-1. For phasing, crystals were soaked in a solution composed of the reservoir solution supplemented with 3 mM potassium tetrachloroplatinate(II). Data were collected at the absorption threshold of Platinum (1.0716 Å).

Native and SAD datasets were indexed using the program XDS [53], and experimental phasing and molecular replacement were carried out with the program Autosol and Phaser [54] from PHENIX [55] and described elsewhere (Loc'h et al. in preparation). Initial rebuilding was carried out with Buccaneer from the CCP4 program suite [56] and subsequent rebuilding and refinement with COOT [57] and the Refine module from PHENIX. The ATP crystal form was solved by molecular replacement with the ADP bound model of the complex using PHENIX. PDB coordinates were deposited with codes 4cwn and 4cw7 for the ADP- and ATP-bound structures.

### SAXS

SAXS data were collected on Beamline SWING at the Soleil Synchrotron at an electron energy of 13.32 keV. Protein samples were centrifuged at 13,200 *g* for 10 min before data acquisition, and concentration was measured with Nanodrop spectrophotometer (Thermo). Two modes of data collection were used: direct injection and size exclusion chromatography separation prior to data collection. For direct injection, samples at known concentration were loaded at a flow rate of 200 µL min^−1^ in the SAXS capillary flow cell. For size exclusion chromatography, samples were loaded on an Agilent SEC-3 size-exclusion chromatography column connected to an Agilent HPLC system at a flow rate of 200 µL min^−1^. In each case, 100 frames, with exposure times of 1 s per frame, were collected at 288 K in the flow cell connected to the HPLC system. The same buffer was used for all data collection (Tris-HCl 50 mM, NaCl 200 mM, pH 7.5). Buffer scattering was collected in the same conditions or using the gel filtration profile before the void volume.

Data reduction—that is, image conversion to 1D profile, absorption correction, scaling to absolute scale, buffer subtraction, averaging, and peak analysis—was carried out using the program FOXTROT, available on Beamline [58]. Further processing and data analysis was done using the programs of the ATSAS suite [59]. Guinier plot analysis was performed using PRIMUS [60] with scattering data at low angle regions with qmax*Rg<1.3. Pair distribution function was calculated with GNOM [61].

*Ab initio* analysis was performed with DAMMIF [62]. The 50 DAMMIF calculations were averaged with DAMAVER [63] to produce averaged and filtered shape. Homology models were made with MODELLER [64]. Modeling with SAXS data was done with DADIMODO [65]. Analysis of the fit between model and experimental data was done with CRYSOL [66]. The χ2 value (Chi) against the experimental SAXS profile and the residual are computed with the Crysol program and defined as: 
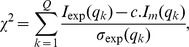

*q_k_* is the momentum transfer, *I_exp_* the experimental intensity data, *I_m_* the intensity calculated from the model, *σ_exp_* the experimental error, and *c* a scaling parameter.

The residual is defined as: 
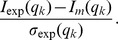



The data collection and processing statistics are reported in Table 2.

### RNA Binding and ATPase Activity Assays

A kinetic enzyme-coupled method for assay of yFap7 AK activity was carried out as previously described for hFap7 [12]. AK and ATPase assays were performed on a safas UV/Vis spectrophotometer. When ATP was used as the substrate, the production of ADP is coupled to β-NADH oxidation via the action of pyruvate kinase (PK) and lactate dehydrogenase (LDH). The rate of β-NADH disappearance was monitored at 340 nm over a 15-min period and at 30°C. Reference samples, containing reaction mixture without yFap7, were used to subtract background absorbance mainly attributable to nonenzymatic ATP hydrolysis and the ATPase activity of puryvate kinase.

The final assay mixture (200 µL) consisted of 100 mM Tris-HCl pH 7.5, 60 mM KCl, 5 mM MgCl2, 0.2 mM β-NADH, 1 mM PEP (Phospho(enol)pyruvate—Sigma), 10 U/mL PK (Sigma), 14 U/ml LDH (Sigma), H_2_O, and 0.01–1 mM ATP. The AK activity of yFap7 (5–10 µM) with respect to ATP was measured in the presence of 0.3 mM AMP. Similar approaches have been carried out to study the ATPase activity of yFap7, the AK activity of yFap7 with respect to AMP, and the AK activity of yFap7 associated with yRps14.

### RNA Binding Assays

Filter binding assays were performed and adapted as described [36]. The labeled RNA was heated at 65°C for 2 min and immediately placed on ice for 10 min and diluted in binding buffer containing 30 mM Tris-HCl pH 8.0, 150 mM KCl, 6 mM MgCl2, and 60 µg of *E. coli* tRNA per ml. Binding reactions consisted of 12 µl of RNA (10 fmol) and 8 µl of proteins (final concentration from 0.0036 to 3.6 µM). Binding reactions were incubated at 20°C for 15 min and then applied directly to filters containing the two membranes under gentle vacuum. Before and after application of the binding reactions, 200 µl of binding buffer without tRNA was used to equilibrate and rinse the system. Binding was quantified using a Molecular Dynamics phosphorimager and Image Gauge program (Fujifilm). The Intensity was corrected for background and fit for *K_d_* using GNUPLOT (Thomas Williams, Colin Kelley et al., http://www.gnuplot.info) using the following equation: 
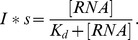



The scale factor *s* was fitted along with Kd for the Rps14/RNA lane and applied to all other measurements.

### Pull-Down Assays

To test interaction between yRps14, yFap7, and RNA, 30 µl of GST-yRps14–coated beads were used for each condition; beads were then resuspended in 1 ml of IP buffer and then treated as described.

Recombinant His-yFap7 was added and incubated for 1 h at 4°C; then beads were washed three times in IP buffer and one time in PBS. When nucleotides were used, they were incubated at the same time at 4°C for 1 h and then at room temperature (RT) for another 15 min before the washes. When both RNA and Fap7 were tested, RNA was first incubated with GST-yRps14+ beads for 1 h at 4°C, then His-yFap7 (with or without nucleotides) was added for another 1 h at 4°C, and then it was incubated at RT for 15 min before being washed.

For protein analysis, beads were resuspended in 50 µl of SDS loading buffer and boiled at 95°C for 5 min, and the supernatant was loaded on SDS-PAGE. For RNA analysis, RNAs were immediately extracted as described previously [4] and analyzed on 15% acrylamide–8 M Urea gel and stain with SYBR Safe.

### Analytical Gel Filtration

Analytical gel filtration experiments for the detection of protein–protein interactions were carried out using an analytical Superdex S200 (GE) gel filtration column with a flow rate of 0.5 ml/min on an Äkta purifier system (GEHealthcare) in a 50 mM Hepes pH 7.5, 300 mM KCl buffer at 20°C. The protein concentrations were 20 µM and the total applied sample volume was 100 µl in all cases. Protein elution was followed by recording the UV adsorption at 280 nm.

### In Vitro Ribosome Maturation Assay

WT, PTH-NOB1 or ΔFAP7, PTH-NOB1 cells were grown in 1 l of YNB glucose (2%) for 8 h to an OD600 of 0.5, collected, and washed with phosphate-buffered saline. Extracts were prepared in 500 µl of buffer A [50 mM Tris pH 7.5, 150 mM NaCl, 5 mM MgCl_2_, 0,1% NP-40, 1 mM DTT, and protease inhibitors (Roche)] using Zirconia beads as previously described [4]. Aliquots corresponding to 12 mg of proteins were added to 50 µl of immunoglobulin G (IgG)-sepharose beads (GE Healthcare) in a 1 ml final volume of buffer A. Immunoprecipitation was performed at 4°C for 1.5 h. Beads were then washed three times (5 min per wash) with 1 ml of buffer A at 4°C. Most of the supernatant was discarded and 50 µl of buffer X (50 mM Tris pH 7.5, 150 mM NaCl, 5 mM Mn2+, 0.1% NP-40, 1 mM DTT, 10% glycerol) added to the pellet (recovered by resuspension in 20 µl of remaining buffer A) to reach a final volume of 70 µl. We added 250 pmoles of recombinant yFap7 or yFap7–yRps14 complex when indicated and incubated it for 20 min at 20°C. Nucleotides were added when required at a final concentration of 1 mM. Reactions were incubated at 20°C for 30 min, and RNAs were then immediately extracted as described [4].

## Supporting Information

Figure S1 Multiple sequence alignment of Fap7 and Rps14 from Archaea, yeast, and human. The sequences of Fap7 and Rps14 from *P. abyssii*, *S. cerevisia*e, and *Homo sapiens* were aligned using T-coffee [67] and rendered using ESPRIPT [68].
(PNG)

Figure S2 Cα contact of the aFap7–aRps14 complex. Distances in Å between Cα atoms of Rps14 and Fap7 were calculated on a 2D matrix and color coded as a function of distance. This simplified representation shows the interaction of the different structural elements, represented with the same color code as Figure 1.
(PNG)

Figure S3 Rps14 uses the same surface for Fap7 and RNA binding. Rps14 residues mapped on the surface of the aRps14 protein in complex with aFap7. aRps14 residues contacting aFap7 are colored green (left) and yRps14 residues contacting rRNA in the ribosome are colored purple (right).
(TIFF)

Figure S4 (A) AK activities of protein aFap7, aRps14, aFap7–aRps14, and aFap7–aRps14ΔC were followed at 37°C ([ATP] = 0.8 mM and [AMP] = 0.3 mM) by a coupled enzyme assay. (B) To test the interaction between yFap7 and ATP, 18 µl of recombinant yFAP7-His (30 µM) was incubated during 5 min with ATPγ ^32^P (0.1 mM) in a final volume of 20 µl. The interaction was tested in the absence and presence of 5 mM MgCl_2_ and with addition of AMP-PNP at 0.1 mM and 1 mM as the final concentrations. Interactions were analyzed on 8% acrymamide-bis-acrylamide native gel, and binding was revealed and quantified using a Molecular Dynamics Phosphoimager and Image Gauge Program (Fujifilm). (C) Betascope image of nitrocellulose filters and 10 fmol per well of helix 23 18S rRNA was incubated with yRps14 (a), yRps14–yFap7 complex (b), and yFap7 (c). Wells 2–8 contained progressively higher concentrations of proteins in steps ranging from 0.0036 to 3.6 µM. (D) Filter binding assays. yFap7 competes with RNA (radiolabeled Helix 23 of 18S rRNA) for yRps14 binding using yRps14-MBP and yFap7-His proteins. Binding buffer contains 20 mM HEPES pH 8.0, 300 mM KCl, 5 mM MgCl2, and binding reactions consisted in 2 µL of RNA (10 fmol) and 18 µL of yRps14-MBP alone or a mix of yRps14-MBP–yFap7-His (from 0.1 µM to 30 µM). Reactions were incubated for 15 min at 20°C before application on the nitrocellulose and nylon membranes. (E) Quantifications of betascope image in panel D. yRps14-MBP binds the RNA with an apparent affinity of 34.9±0.95 µM and the complex formed by mixing yRps14-MBP and yFap7-His in stoichiometric concentrations with a lower affinity of 82.5±4.4 µM. (F and G) We used 10 µL of each filter binding reaction (panel D) for RNA extraction as described in Materials and Methods. RNAs were analyzed on denaturing 8% acrylamide–8M urea gel and visualized using the phosphoimaginer. We quantified each band corresponding to the helix 23 of 18S rRNA to verify that there is no degradation of RNA with addition of protein during the filter binding assays with yRps14 (F) and yRps14–yFap7 (G). (H) To identify proteins associated to PTH-Nob1 during our *in vitro* maturation assay, we performed a mass spectrometry analysis. Extracts used for *in vitro* maturation assay were prepared as described for the test, except that instead of being incubated at 30°C, beads were resuspended in SDS loading buffer and boiled at 95°C for 5 min. Supernatant issued from WT, PTH-Nob1, or Gal::Fap7,PTH-Nob1 cells was loaded on SDS-PAGE gel for a short migration. (I) Variation of cleavage efficiencies in relation to nucleotide addition. Signals obtained for cleavage at site D in panel A were quantified, corrected for RNA loading (using a common stop in the ITS1 as a standard), and normalized to the mock-treated sample (set to 1). The average of two independent experiments is shown, with the standard deviation indicated on top of the histogram.
(TIF)

Figure S5 Dissociation assay with the co-expressed Fap7–Rps14 complex. Effects of ATP, ADP, and Amppnp on binding of the HIS-yFap7–yRps14 complex used for co-crystallizations were tested by pulldown experiment in the presence of 5 mM MgCl_2_.
(PNG)

Movie S1 Animation of the proposed conformational transition leading to Rps14 release. A conformational transition was modeled by extrapolating the movement between three conformations of the Fap7–Rps14 complex. This movement models the molecular events leading to the release of Rps14 from Fap7. This conformation transitions in the LID and NMP domains of Fap7 and of the C-terminal extension and β4–α2 loop of Rps14.
(MPG)

Table S1 Mass spectroscopy analysis of proteins associated with nob1 particles used for maturation assay. Proteins were cut from the gel and subsequently treated for mass spectrometry analysis. We identified 89 and 86 proteins for “WT” or “Gal::Fap7” samples, respectively. Only proteins with at least two peptides were conserved for the analysis. Proteins considered as common yeast IP contaminants were also discarded [69].
(PDF)

## Abbreviations

ADP: adenosine di-phosphate
AK: adenylate kinase
AMP: adenosine mono-phosphate
AMPK: AMP-activated protein kinase
Ap5A: diadenosine pentaphosphate
ATP: adenosine nucleotide triphosphate
DTT: Dithiothreitol
IMAC: metal ion affinity chromatography
LDH: lactate dehydrogenase
PEP: Phospho(enol)pyruvate
PK: pyruvate kinase
Rg: radius of gyration
r.m.s.d.: root mean square deviation
RNP: ribonucleoprotein
RT: room temperature
SAXS: small angle X-ray scattering
SDS-PAGE: sodium dodecyl sulfate polyacrylamide gel electrophoresis
snRNP: small nuclear ribonucleoprotein
