# Supplementary figures and images for: RNA Mimicry by the Fap7 Adenylate Kinase in Ribosome Biogenesis
Source: PLoS Biol. 2014 May 13;12(5):e1001860. doi: 10.1371/journal.pbio.1001860 (PMC4019466; doi:10.1371/journal.pbio.1001860)

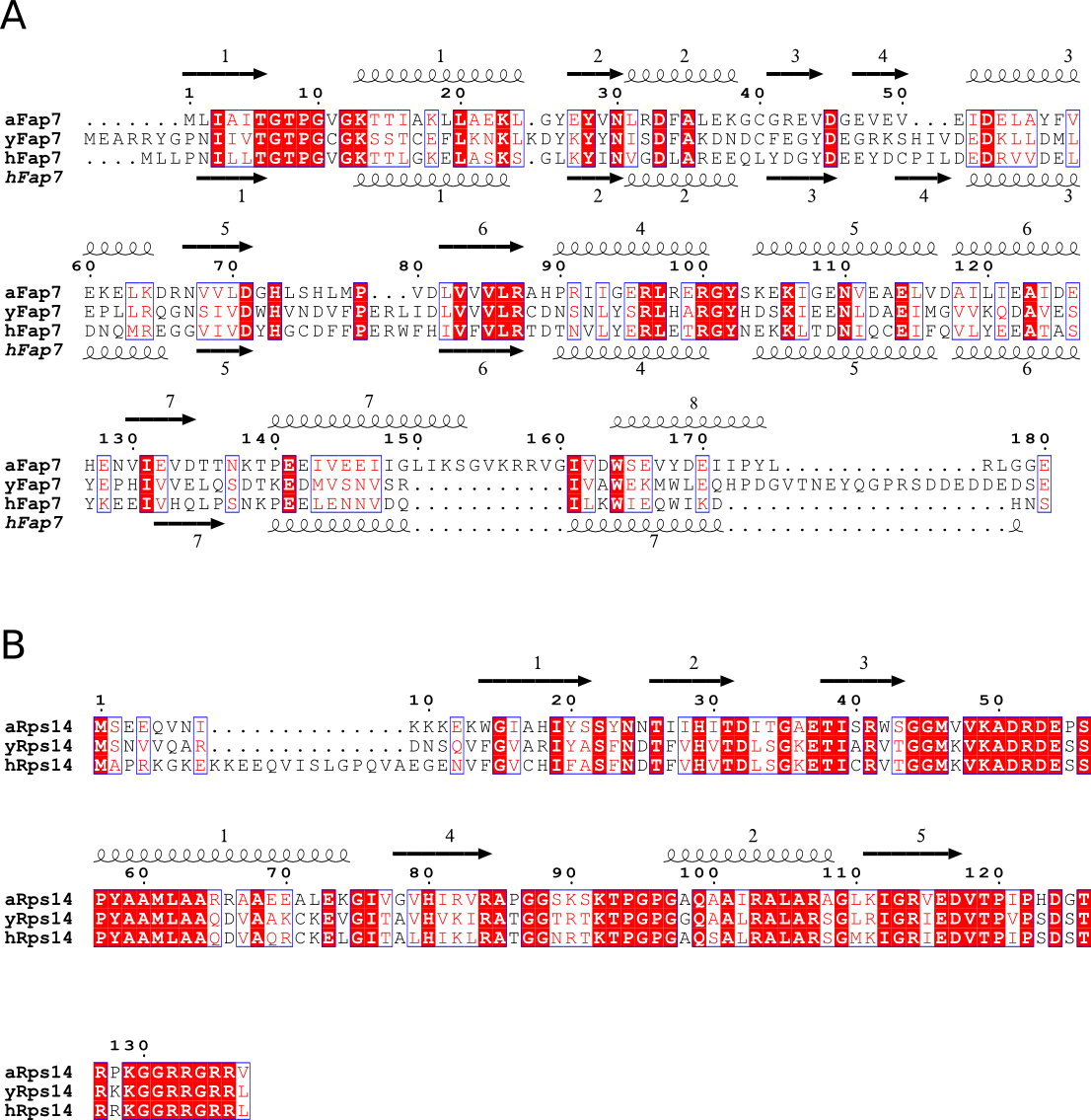

Supplement: Figure S1 — Multiple sequence alignment of Fap7 and Rps14 from Archaea, yeast, and human. The sequences of Fap7 and Rps14 from P. abyssii, S. cerevisiae, and Homo sapiens were aligned using T-coffee [67] and rendered using ESPRIPT [68]. (PNG) [file pbio.1001860.s001.png]

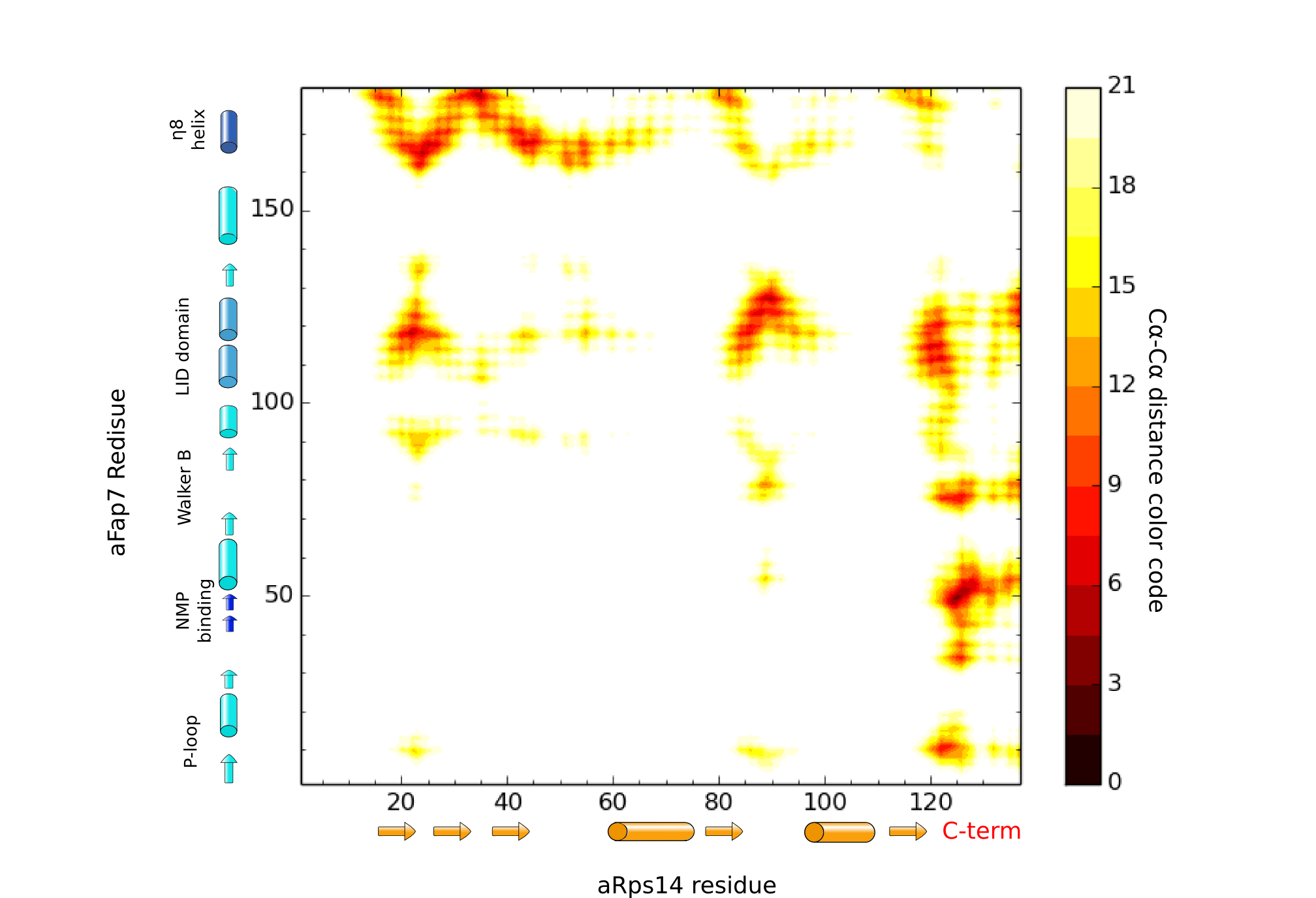

Supplement: Figure S2 — Cα contact of the aFap7–aRps14 complex. Distances in Å between Cα atoms of Rps14 and Fap7 were calculated on a 2D matrix and color coded as a function of distance. This simplified representation shows the interaction of the different structural elements, represented with the same color code as Figure 1. (PNG) [file pbio.1001860.s002.png]

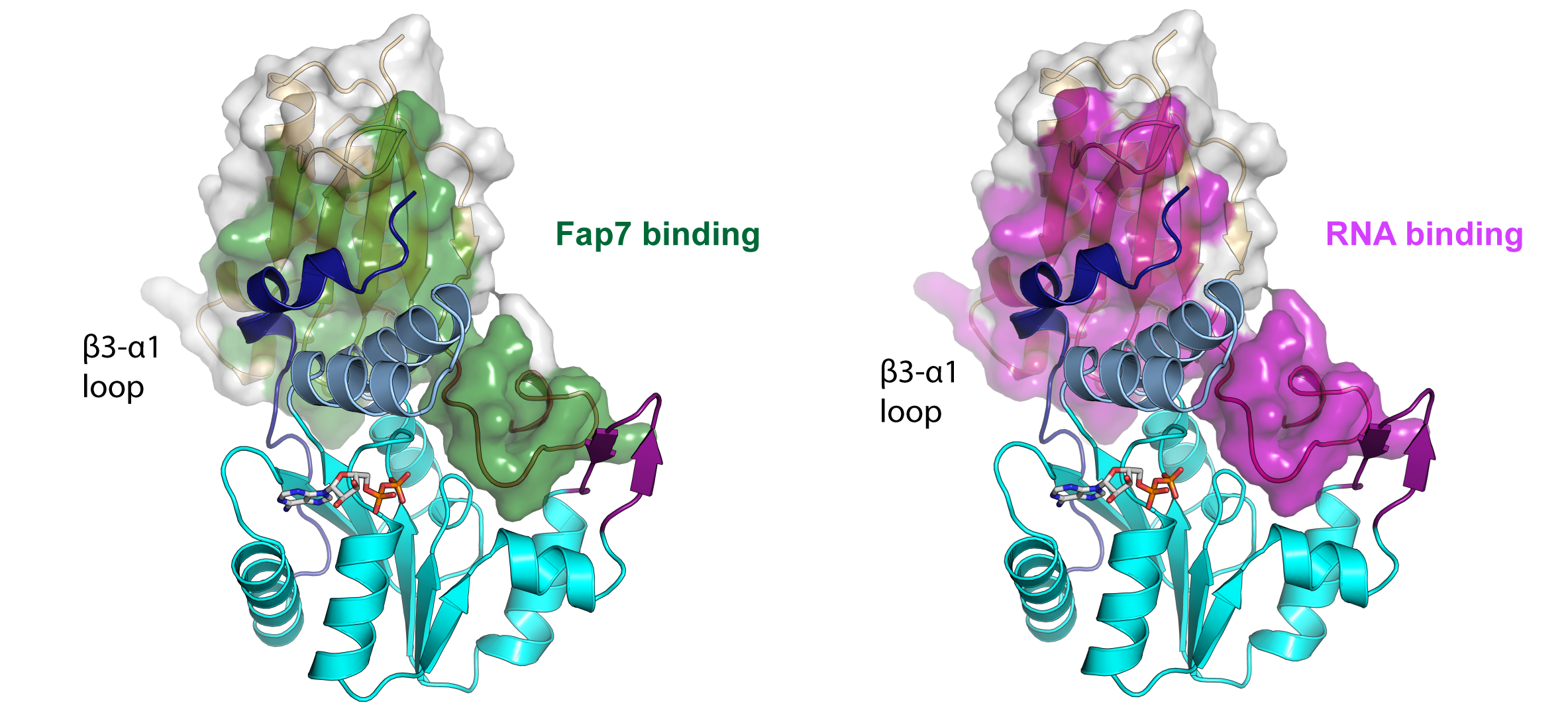

Supplement: Figure S3 — Rps14 uses the same surface for Fap7 and RNA binding. Rps14 residues mapped on the surface of the aRps14 protein in complex with aFap7. aRps14 residues contacting aFap7 are colored green (left) and yRps14 residues contacting rRNA in the ribosome are colored purple (right). (TIFF) [file pbio.1001860.s003.tif]

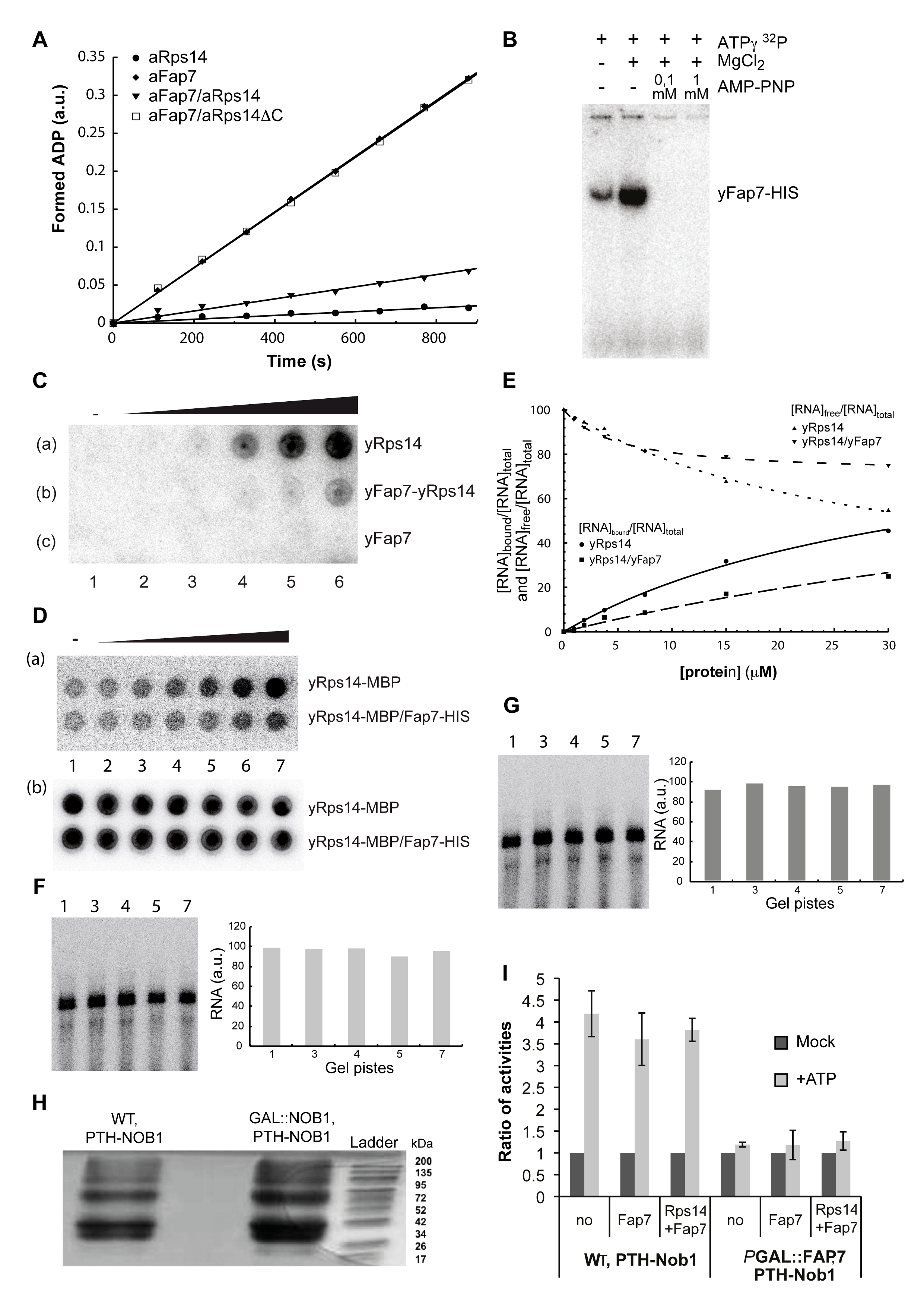

Supplement: Figure S4 — (A) AK activities of protein aFap7, aRps14, aFap7–aRps14, and aFap7–aRps14ΔC were followed at 37°C ([ATP] = 0.8 mM and [AMP] = 0.3 mM) by a coupled enzyme assay. (B) To test the interaction between yFap7 and ATP, 18 µl of recombinant yFAP7-His (30 µM) was incubated during 5 min with ATPγ 32P (0.1 mM) in a final volume of 20 µl. The interaction was tested in the absence and presence of 5 mM MgCl2 and with addition of AMP-PNP at 0.1 mM and 1 mM as the final concentrations. Interactions were analyzed on 8% acrymamide-bis-acrylamide native gel, and binding was revealed and quantified using a Molecular Dynamics Phosphoimager and Image Gauge Program (Fujifilm). (C) Betascope image of nitrocellulose filters and 10 fmol per well of helix 23 18S rRNA was incubated with yRps14 (a), yRps14–yFap7 complex (b), and yFap7 (c). Wells 2–8 contained progressively higher concentrations of proteins in steps ranging from 0.0036 to 3.6 µM. (D) Filter binding assays. yFap7 competes with RNA (radiolabeled Helix 23 of 18S rRNA) for yRps14 binding using yRps14-MBP and yFap7-His proteins. Binding buffer contains 20 mM HEPES pH 8.0, 300 mM KCl, 5 mM MgCl2, and binding reactions consisted in 2 µL of RNA (10 fmol) and 18 µL of yRps14-MBP alone or a mix of yRps14-MBP–yFap7-His (from 0.1 µM to 30 µM). Reactions were incubated for 15 min at 20°C before application on the nitrocellulose and nylon membranes. (E) Quantifications of betascope image in panel D. yRps14-MBP binds the RNA with an apparent affinity of 34.9±0.95 µM and the complex formed by mixing yRps14-MBP and yFap7-His in stoichiometric concentrations with a lower affinity of 82.5±4.4 µM. (F and G) We used 10 µL of each filter binding reaction (panel D) for RNA extraction as described in Materials and Methods. RNAs were analyzed on denaturing 8% acrylamide–8M urea gel and visualized using the phosphoimaginer. We quantified each band corresponding to the helix 23 of 18S rRNA to verify that there is no degradation of RNA with addition of pr [file pbio.1001860.s004.tif]

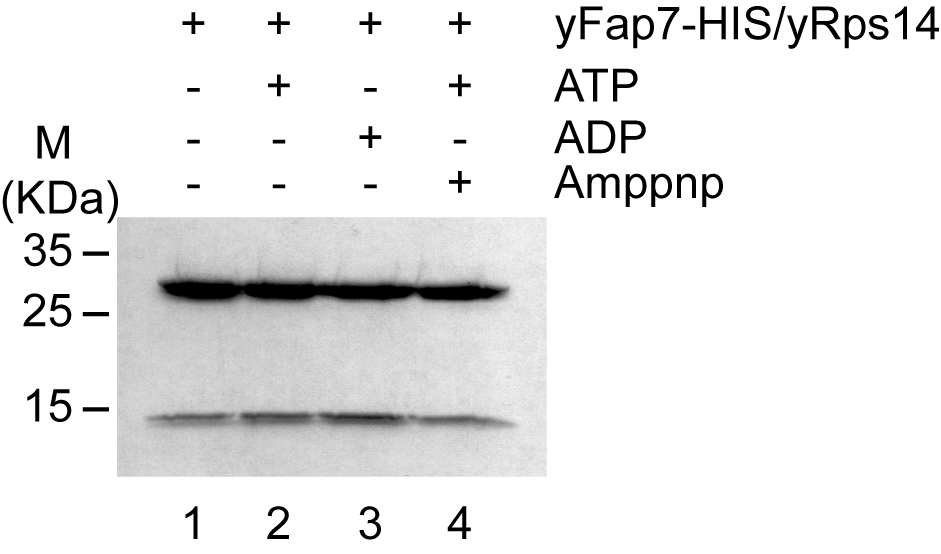

Supplement: Figure S5 — Dissociation assay with the co-expressed Fap7–Rps14 complex. Effects of ATP, ADP, and Amppnp on binding of the HIS-yFap7–yRps14 complex used for co-crystallizations were tested by pulldown experiment in the presence of 5 mM MgCl2. (PNG) [file pbio.1001860.s005.png]
