# Supplementary material for: RNA Mimicry by the Fap7 Adenylate Kinase in Ribosome Biogenesis
Source: PLoS Biol. 2014 May 13;12(5):e1001860. doi: 10.1371/journal.pbio.1001860 (PMC4019466; doi:10.1371/journal.pbio.1001860)
Supplement: Table S1 — Mass spectroscopy analysis of proteins associated with nob1 particles used for maturation assay. Proteins were cut from the gel and subsequently treated for mass spectrometry analysis. We identified 89 and 86 proteins for “WT” or “Gal::Fap7” samples, respectively. Only proteins with at least two peptides were conserved for the analysis. Proteins considered as common yeast IP contaminants were also discarded [69]. (PDF) [file pbio.1001860.s007.pdf]

Proteins	WT, PTH-NOB1[i]	GAL::FAP7, PTH-NOB1[a]	Delta[ii]
40S ribosomal proteins			
**Rps8a**	+	-	-100
**Rps1a**	++	+	-63.6
**Rps1b**	++	+	-60
**Rps6a**	+	+	-20
**Rps9a**	+++	++	-15.8
**Rps9b**	+++	++	-11.8
**Rps7a**	++	++	-5.9
**Rps5**	+++	+++	-4.8
**Rps7b**	++	++	0
**Rps4a**	++	++	5.3
**Rps18a**	++	++	14.3
**Rps19a**	+	+	20
**Rps3**	++	+++	29.4
**Rps12**	+	++	33.3
**Rps21a**	+	+	33.3
**Rps17a**	+	++	55.6
**Rps24a**	+	++	55.6
**Rps20**	+	++	75
**Rps13**	-	+	100
**Rps15**	-	+	100
**Rps16a**	-	+	100
60S ribosomal proteins			
**P0**	+	+	-14.3
**Rpl12a**	+	+	0
**Rpl8a**	++	++	6.7
**Rpl8b**	++	++	12.5
**Rpl13a**	+	+	20
**Rpl17a**	+	++	20
**Rpl4a**	++	++	23.1
**Rpl1a**	+	+	33.3
**Rpl21a**	+	+	33.3
**Rpl5**	+	++	60
**Rpl10**	-	+	100
**Rpl16b**	-	+	100
**Rpl20a**	-	+	100
**Rpl25a**	-	+	100
**Rpl26b**	-	++	100
90S accessory factors			
**Bud22**	+	-	-100
**Cms1**	+	-	-100
**Enp2**	+	-	-100
**Fcf2**	++	-	-100
**Imp3**	++	-	-100
**Mpp10**	++	-	-100
**Nop13**	+	-	-100

Proteins	WT, PTH-NOB1[a]	GAL::FAP7, PTH-NOB1[a]	Delta[b]
**Sof1**	+	-	-100
**Utp11**	+	-	-100
**Utp19**	+	-	-100
**Utp3**	++	-	-100
**Bfr2**	++	+	-77.8
**Utp2**	++	+	-75
**Imp4**	+	+	-60
**Nop56**	+++	+	-57.1
**Nop58**	++	+	-50
**Nop6**	++	+	-50
**Utp9**	++	+	-50
**She1**	++	+	-40
**Kri1**	+	+	-33.3
**Pwp1**	+	+	-33.3
**Utp15**	+	+	-33.3
**Snu13**	+	+	-20
40S accessory factors			
**Krr1**	+	-	-100
**Ltv1**	+++	-	-100
**Rio1**	+	-	-100
**Rio2**	+++	-	-100
**Tsr1**	+	-	-100
**Enp1**	+++	+	-76.5
**Pno1**	++	+	-25
**Nob1**	++	++	6.7
60S accessory factors			
**Nop12**	+	-	-100
**Rrp1**	+	-	-100
Others			
**Pab1**	+++	-	-100
**Adh1**	+	+	0
**Npl3**	+	+	0
**Efb1**	++	++	9.1
**Mpd2**	+	+	14.3
**Stm1**	+	++	25
**Dbp2**	+	+	33.3
**Yra1**	+	+	50

[i] "-" and "+" signs represent the number of peptides for each proteins:
- =0; += 0 to 5; ++= 5 to 10; +++=10 or more.

[ii] Delta represents the different between the numbers of peptides as percentage of the total number of peptides. Positive and negatives values indicate the sense of the displacement of the equilibrium.
